# Isatuximab Acts Through Fc-Dependent, Independent, and Direct Pathways to Kill Multiple Myeloma Cells

**DOI:** 10.3389/fimmu.2020.01771

**Published:** 2020-08-14

**Authors:** Chen Zhu, Zhili Song, Anlai Wang, Srimathi Srinivasan, Guang Yang, Rita Greco, Joachim Theilhaber, Elvis Shehu, Lan Wu, Zhi-Yong Yang, Wilfried Passe-Coutrin, Alain Fournier, Yu-Tzu Tai, Kenneth C. Anderson, Dmitri Wiederschain, Keith Bahjat, Francisco J. Adrián, Marielle Chiron

**Affiliations:** ^1^Sanofi Oncology, Cambridge, MA, United States; ^2^Sanofi Research and Development, Sanofi North America, Cambridge, MA, United States; ^3^Sanofi R&D, Biomarkers and Clinical Bioanalyses, Paris, France; ^4^Sanofi R&D, Tumor-Targeted Immuno-Modulation I, Vitry-sur-Seine, France; ^5^Jerome Lipper Multiple Myeloma Center, LeBow Institute for Myeloma Therapeutics, Dana-Farber Cancer Institute, Harvard Medical School, Boston, MA, United States

**Keywords:** antibody-dependent cellular cytotoxicity, antibody-dependent cellular phagocytosis, CD38, isatuximab, natural killer cells, multiple myeloma, PD-1, TGF-β

## Abstract

Isatuximab is a monoclonal antibody targeting the transmembrane receptor and ectoenzyme CD38, a protein highly expressed on hematological malignant cells, including those in multiple myeloma (MM). Upon binding to CD38-expressing MM cells, isatuximab is thought to induce tumor cell killing via fragment crystallizable (Fc)-dependent mechanisms, including antibody-dependent cellular cytotoxicity (ADCC), antibody-dependent cellular phagocytosis (ADCP), and complement-dependent cytotoxicity (CDC), as well as via direct Fc-independent mechanisms. Here, these mechanisms of action were investigated in MM and diffuse large B-cell lymphoma (DLBCL) cell lines, as well as in peripheral blood mononuclear cells derived from healthy donors, and in MM patient-derived samples. Our findings show that isatuximab-mediated cytotoxicity occurred primarily via ADCC and ADCP in MM cell lines and via ADCC and apoptosis in DLBCL cell lines expressing high levels of CD38. We identified the programmed cell death-1/programmed cell death-ligand 1 (PD-1/PD-L1) pathway and MM cell-secreted transforming growth factor-beta (TGF-β) as tumor cell-related features that could suppress CD38-mediated ADCC. Furthermore, we established that isatuximab can directly activate natural killer (NK) cells and promote NK cell-mediated cytotoxicity via crosslinking of CD38 and CD16. Finally, isatuximab-induced CDC was observed in cell lines with high CD38 receptor density (>250,000 molecules/cell) and limited expression of inhibitory complement regulatory proteins (CD46, CD55, and CD59; <50,000 molecules/cell). Taken together, our findings highlight mechanistic insights for isatuximab and provide support for a range of combination therapy approaches that could be tested for isatuximab in the future.

## Introduction

CD38 is a type II transmembrane protein expressed on a variety of immune cells (1, 2). CD38 functions both as a receptor, impacting processes such as leukocyte migration and activation (2–4), and as a multifunctional ectoenzyme, modulating calcium signaling (2). Under physiological conditions, CD38 is expressed at low levels on immune cells (1), while plasma cells in both healthy individuals and patients with multiple myeloma (MM) show significantly higher CD38 expression (5, 6). Elevated expression of CD38 is also found in other hematological malignancies such as germinal center B-like diffuse large B-cell lymphoma (GCB-DLBCL) (7) and its expression has been linked to prognosis in chronic lymphocytic leukemia (8), acute lymphocytic leukemia, and acute myeloid leukemia (9, 10). Therefore, CD38 is an appealing tumor-associated antigen for targeted therapy; and monoclonal antibodies against CD38, such as daratumumab, have shown efficacy in the clinical treatment of relapsed and refractory multiple myeloma (RRMM) and newly diagnosed MM patients (11–13).

Isatuximab is an immunoglobulin G (IgG)1 monoclonal antibody that targets a specific epitope on CD38 (14). The Phase 3 ICARIA-MM trial (NCT02990338) evaluated isatuximab, in combination with pomalidomide and dexamethasone, in patients with RRMM who had received at least two prior lines of treatment, including lenalidomide and a proteasome inhibitor (15). The addition of isatuximab to pomalidomide and dexamethasone significantly improved progression-free survival and was well tolerated (15). The ICARIA-MM study has led to the approval of isatuximab in the US, EU, Switzerland, Canada, and Australia, in combination with pomalidomide and dexamethasone, for the treatment of adult patients with MM who have received at least two prior therapies including lenalidomide and a proteasome inhibitor (16–19).

A number of studies have investigated the mechanism of action of isatuximab, identifying both fragment crystallizable (Fc)-dependent and Fc-independent activities leading to killing of CD38-expressing tumor cells. Fc-dependent mechanisms induced by isatuximab comprise antibody-dependent cellular cytotoxicity (ADCC), antibody-dependent cellular phagocytosis (ADCP), and complement-dependent cytotoxicity (CDC) (14, 20–22), with ADCC suggested to be the dominant effector mechanism (20, 21). ADCC is mediated by activating Fcγ receptors on the surface of natural killer (NK) cells binding to the Fc regions of IgG. This is thought to lead to killing of antibody-bound target cells via perforin and granzyme released from NK cells, and tumor necrosis factor (TNF) family receptor signaling (23). The release of proinflammatory cytokines as a result of ADCC can also promote downstream immune cell activation (23). However, tumor cells may suppress immune responses through different mechanisms including the programmed cell death-1 (PD-1)/programmed cell death-ligand 1 (PD-L1) axis (24). Indeed, there is evidence for the PD-1/PD-L1 pathway regulating the cytolytic activity of NK cells in patients with MM (25). Another mechanism contributing to immunosuppression is the soluble mediator transforming growth factor-beta 1 (TGF-β1), which has been shown to suppress ADCC (26). As well as ADCC, CD38 ligation may also lead to direct activation of NK cell cytotoxicity (27), mediated by signaling via CD16 (FcγRIIIa) (28).

ADCP mediated by macrophage Fcγ receptor binding to the antibody Fc regions and leading to phagocytosis of the antibody-bound cell has been described to play a major role in the antitumor activity of therapeutic antibodies (29). Complement pathway activation following deposition of the complement component C1q to Fc regions leads to target cell death through the deposition of opsonins, such as C3b, onto target cell surfaces, promoting phagocytosis, as well as the insertion of a membrane attack complex into the target cell membrane, ultimately resulting in direct cell lysis (30). The consequences of the complement pathway activation can be counteracted by several regulatory mechanisms to protect normal tissues, including complement regulatory proteins such as CD46, CD55, and CD59 (30). Upregulation of these CDC inhibitor proteins within tumor cells might protect them from CDC-mediated lysis (30).

Fc-independent activities could play important roles in isatuximab-mediated killing of CD38-expressing MM cells. Isatuximab induces direct cytotoxicity via caspase-dependent and lysosome-associated pathways in MM cells (31), and also inhibits the ADP ribosyl-cyclase ectoenzymatic activities of CD38 (14). Interestingly, adenosine generated in the bone marrow niche via CD38-mediated pathway is correlated with progression of myeloma (32); thus, this mechanism of isatuximab might contribute to antitumor immunity in this environment.

Multiple mechanisms of action likely contribute to the clinical activity of isatuximab in patients with MM (21, 31). Delineating these mechanisms could help to identify patients who are most likely to respond to treatment, as well as suggesting complementary pathways to target in combination therapy (21). In this study, we investigated the mechanisms of action of isatuximab, in terms of ADCC, ADCP, CDC, the induction of apoptosis, and direct NK cell activation, using a panel of established MM and DLBCL cell lines, peripheral blood mononuclear cells (PBMCs) from healthy human donors, and MM patient samples.

## Materials and Methods

### Cell Lines, Human Blood Samples, and MM Patient Samples

All MM and DLBCL cell lines used were purchased from American Type Culture Collection (ATCC; Manassas, VA), Deutsche Sammlung von Mikroorganismen und Zellkulturen (DSMZ; Braunschweig, Germany), and Health Science Research Resources Bank (HSRRB; Osaka, Japan) and cultured according to suppliers' recommendations (typically RPMI 1664 with 10% or 20% fetal bovine serum [FBS]). Supplementary Table 1 presents information regarding the cell lines used from the Cancer Cell Line Encyclopedia (33). CD38-overexpressing cell lines (RPMI-8226.CD38^++^, JJN-3.CD38^++^, U-266.CD38^++^, OCI-LY19-CD38^++^, and NCI-H929.CD38^++^) were generated by lentiviral transduction of full-length human CD38 complementary DNA (Sanofi R&D) and cultured according to suppliers' recommendations plus 10, 3, 1, 20, or 10 μg/ml blasticidin, respectively. K562 cells were obtained from the European Collection of Authenticated Cell Cultures (Porton Down, UK) and were cultured in Iscove Modified Dulbecco Media (Invitrogen, Carlsbad, CA); JHH-6 cells were obtained from the HSRRB and were cultured in William's E medium (Gibco; Thermo Fisher Scientific, Waltham, MA); and THP-1 cells were obtained from the ATCC and were cultured in RPMI 1640 medium (Gibco). NK-92 parental cells, and stable cell lines expressing different variants of CD16, CD16^F/F^ (NK-92.CD16^F/F^, low-affinity CD16 variant), and CD16^V/V^ (NK-92.CD16^V/V^, high-affinity CD16 variant), were purchased from Conkwest, Inc. (Cardiff-by-the-Sea, CA) and were maintained in the suppliers' recommended media (MyeloCult H5100 [STEMCELL Technologies, Vancouver, Canada; cat #05150] plus recombinant human interleukin (IL)-2, 100 units/ml (R&D Systems, Minneapolis MN; cat #202-IL-010/CF).

All human PBMCs were isolated from buffy coats of healthy donors (Massachusetts General Hospital, Boston, MA) by Ficoll-Paque (GE Healthcare, Chicago, IL) density gradient centrifugation according to the manufacturer's protocol. Human NK cells, monocytes, T cells, and B cells were purified from PBMCs using relevant EasySep™ Enrichment Kits (STEMCELL Technologies) by negative selection according to the manufacturer's instructions. Primary NK cells were cultured in Myelocult H5100 complete medium (STEMCELL Technologies) and IL-2 (100 units/ml). MM patient samples were obtained after informed consent was provided, in accordance with the Declaration of Helsinki and under the auspices of a protocol approved by the Institutional Review Board of the Dana-Farber Cancer Institute (Boston, MA). Primary patient MM cells were purified using anti-CD138 microbeads (Miltenyi Biotech, Auburn, CA) as previously described (10, 12, 20, 31).

### Antibodies and Reagents

The anti-TGF-β antibody (1D11; neutralizes TGF-β1,2,3), IgG1 isotype control antibody (13C4), anti-PD-L1 antibody, anti-PD-1 antibody (biosimilar version of nivolumab), isatuximab, isatuximab mutant (Isa^*^), and F(ab')_2_ portion of isatuximab (F(ab')_2_) were generated in-house. Isa^*^ is a mutant of isatuximab unable to bind CD38 because of Y101K and Y102E amino acid substitutions in the variable heavy chain 1 region. The anti-CD16 antibody (cat #302050) was purchased from BioLegend (San Diego, CA). The mouse antihuman CD38 antibody (clone AT13/5) and control mouse IgG for CD38 receptor density testing were from Santa Cruz Biotech (Dallas, TX). Human CD38 was also detected by commercial anti-CD38 monoclonal antibody (clone HB7; Santa Cruz Biotech and BD Biosciences, San Jose, CA). Mouse antihuman CD46 (cat #555948), CD55 (cat #555691), CD59 (cat #555761), PE-conjugated mouse antihuman CD14 antibody (cat #555398), and isotype control antibodies (cat #555571) were purchased from BD Biosciences. For CD59 blockage, antibodies were purchased from AbD Serotec (Raleigh, NC) (rat antihuman CD59, clone YTH53.1, IgG2b [cat #MCA715G] and rat IgG2b negative control [cat #MCA1125]). The following antibodies for Western blotting were purchased from Cell Signaling Technology, Inc. (Danvers, MA): STAT3 (cat #9132), p-STAT3 (Y705) (cat #9131), p-STAT3 (S727) (cat #9134), STAT5 (cat #9363), p-STAT5 (Y694) (cat #9351), Src kinase (cat #2108), p-Src kinase (Y416) (cat #2101), and actin (cat #3700). All antibodies and relevant isotype controls used for flow cytometry were purchased from BioLegend.

Calcein AM and probenecid were purchased from Invitrogen. Dimethyl sulfoxide and Triton X-100 were purchased from Sigma-Aldrich Corporation (St. Louis, MO). Human IgG1, IgG4, human complement serum, Accutase, and PKH67 green fluorescence cell linker kit were purchased from Sigma-Aldrich. AlamarBlue and FBS with ultra-low IgG were from Invitrogen. Fluorescein isothiocyanate (FITC)-conjugated goat IgG fraction to human complement C3 was obtained from MP Biomedicals (Santa Ana, CA). All-trans retinoic acid (ATRA) was purchased from Sigma-Aldrich.

### Flow Cytometry

For cell-surface protein detection, cells resuspended in 100 μl of ice-cold 1× phosphate-buffered saline (PBS) and 1% bovine serum albumin (BSA) with 5 μl of human Fc block (BD Biosciences, cat #564220) to a final concentration of 5 × 10^6^ cells/ml were incubated with fluorochrome-conjugated antibody of interest on ice in the dark for 30 min. After washing twice with 1× PBS and 0.1% BSA, cells were resuspended in 100 μl of wash buffer for fluorescence-activated cell sorting analysis. For detecting dead cells in the sample, cells were incubated with 100 μl of PBS with 1:100 dilution of Zombie NIR dye (BioLegend) for 10 min at room temperature. For intracellular protein detection, cells (with or without surface antibody staining) were pre-stained with Zombie NIR dye, then fixed in 200 μl of fixation buffer (BioLegend) for 20 min in the dark at room temperature. The fixed cells were pelleted by centrifugation and then washed twice with 200 μl of 1× permeabilization buffer (BioLegend) before staining with fluorochrome-conjugated antibody of interest in 100 μl of permeabilization buffer overnight in the dark at 4°C. After washing twice, the cells were resuspended in 100 μl 1× PBS and 0.1% BSA for analysis by flow cytometry (FACSCanto II, Becton Dickinson, Franklin Lakes, NJ). The data were analyzed using FlowJo software (FlowJo LLC, Ashland, OR).

### Surface Receptor Density Determination

Surface densities of CD38, CD46, CD55, and CD59 receptors were quantitatively determined using QIFIKIT (Agilent Technologies, Inc., Santa Clara, CA) per manufacturer's instructions. Briefly, ~0.5 million test cells were incubated in 100 μl of PBS with a mouse primary antibody specific for the target receptor or an isotype control antibody (10 μg/ml) on ice for 30 min. After washing three times with Ca^2+^- and Mg^2+^-free PBS, primary antibody-bound cells or the calibration bead mix were incubated with the FITC-conjugated detection antibody for 30 min on ice. Fluorescence was measured by flow cytometry using a FACSCalibur (Beckton Dickinson) with CellQuest Pro software (v5.2) (BD Biosciences). A calibration curve was constructed by plotting the fluorescence intensity of the individual bead populations against the number of mouse antibody molecules on the beads. The number of antigenic sites on the test cells was then determined using the calibration curve. To determine CD38 surface density on primary MM cells, 2,200 to 180,000 cells were used for each test.

### ADCC Assay

Target MM or DLBCL cells were labeled with 2 μM of calcein AM at 37°C for 30 min and then washed three times with RPMI 1640 phenol red-free medium (Gibco) supplemented with 1% low IgG FBS and 2.5 mM probenecid. 50 μl of each cell suspension containing 6 × 10^4^ cells was transferred into each well of U-bottom 96-well plates (Corning Inc., Corning, NY). Isatuximab and control antibodies (either Isa^*^ or human IgG1) were incubated with the target cells at 37°C for 30 min. As a positive control, 0.5% Triton X-100 solution was added to the cells to reach the maximum release of calcein AM. Calcein AM-labeled tumor cell culture medium was used to measure spontaneous release. In the next step, 50 μl of effector cells (NK92 or primary NK cells) containing 1.8 × 10^5^ cells were mixed with target tumor cells to achieve a 3:1 effector:target cell ratio. When PBMCs were used as effector cells, the effector:target cell ratio was 20-30:1. The total volume of assay reaction was adjusted to 150 μl with complete medium. After a 1-h incubation in a 37°C incubator, 90 μl of each supernatant was collected from the plates and transferred to 96-well black plates with clear bottom (Corning). Endpoint readings were collected using the EnVision multimode plate reader time-resolved fluorescence laser module (Perkin Elmer, Waltham, MA) using the fluorescence mode with excitation 492 nm and emission 515 nm, with agitation. The intensity of the fluorescence signal was directly proportional to the number of lysed cells and is expressed in arbitrary fluorescent units. The cytolytic activities at a given time point were calculated and presented as the percentage of cytolysis (% of cytolysis = experimental lysis – spontaneous lysis)/(maximal lysis – spontaneous lysis) × 100.

### ADCP Assay

Target MM or DLBCL cells were labeled with 2 μM PKH67 for 5 min at room temperature followed by adding an equal volume of 100% low IgG FBS to stop labeling. After washing, 5 × 10^4^-labeled target cells were resuspended in 100 μl of cell culture medium containing 1 μg/ml of isatuximab or isotype control human IgG (hIgG) in round-bottom 96-well plates, and incubated for 20 min at 37°C. 1.5 × 10^5^ THP-1 cells in 100 μl medium were added for co-culture for 1 h at 37°C. Cells were pelleted and treated with 50 μl of Accutase for 15 min at 37°C. Subsequently, washed cells were stained with anti-CD14-PE or isotype control-PE on ice for 30 min. Finally, washed cells were fixed in PBS with 2% paraformaldehyde. Cell-associated fluorochromes were measured by flow cytometry as described in the flow cytometry section. Phagocytosis was calculated as the percentage of PKH67^+^ THP-1 cells out of the total cell population.

### Apoptosis Assay

The PE-annexin V apoptosis detection kit (Becton Dickinson) was used to quantify isatuximab-induced apoptosis in MM and DLBCL cells according to the manufacturer's protocol. Briefly, 2.5 × 10^5^ cells in 250 μl of complete cell culture medium were treated with isatuximab or hIgG at 10 μg/ml in a 96-well plate for 24 h at 37°C. After washing with PBS, cells were resuspended in 100 μl of 1× binding buffer, and 5 μl of PE-annexin V and 7-aminoactinomycin D were added to each well. Cells then were incubated at room temperature for 15 min, and 100 μl of 1× binding buffer was added to each well. Cells were immediately analyzed by flow cytometry as described in the flow cytometry section. The percentage of cells that had been induced to undergo apoptosis was determined by subtracting the percentage of annexin V^+^ cells in the hIgG-treated population from percentage of annexin V^+^ cells in the isatuximab-treated population.

### Cytokine Stimulation and Quantification

To assess the impact of cytokine stimulation on PD-L1 and PD-L2 expression, MM cells were stimulated with IL-6, TGF-β1, or interferon-gamma (IFN-γ) (R&D Systems).

For cytokine quantification, supernatants were thawed on ice. IFN-γ, IL-2, and TNF-α were assessed by mesoscale discovery using the V-PLEX human proinflammatory panel I (4-plex) kit (Meso Scale Diagnostics, Rockville, MD) according to the manufacturer's instructions.

### Cytolysis

K562 cells were labeled with calcein AM, washed as described for the ADCC assay and seeded into a 96-well plate (Corning) at 40,000 cells/well. NK92.CD16^V/V^ or isolated primary NK cells were added at 10:1 effector:target cell ratio. Finally, antibodies (IgG1 or isatuximab) were added at 1 μg/ml. Maximum lysis was prepared using labeled target cells treated with 0.5% Triton X-100. Calcein AM-labeled target cell culture medium without the addition of effector cells and antibody was used to measure spontaneous release. The volume in the plate was adjusted to 150 μl with complete medium and the plate incubated for 1–2 h at 37°C after bringing the cells to the bottom of the plate by centrifuging for 1 min. Detection was as described for the ADCC assay.

### Western Blot

5 × 10^6^ NK-92 cells (parental or CD16^F/F^ or CD16^V/V^) were seeded in six-well plates and serum-starved for at least 24 h before stimulation with 1 μg/ml of the Isa^*^, isatuximab, F(ab')_2_, anti-CD16 antibody, or 30 ng/ml IL-15 for 15 min. Cells were then lysed using the RIPA lysis buffer system (Santa Cruz Biotech) according to manufacturer's instructions. Lysates were centrifuged at 13,000 rpm and the supernatants were used for protein quantification and Western blotting. Protein concentrations were determined by Pierce BCA protein assay kit (Thermo Fisher Scientific) using BSA as a standard. Equal amounts of total protein (5 μg) for each sample were analyzed by Western blot, performed by using Wes split buffer master kit (ProteinSimple, San Jose, CA) on the “Wes” automated Western blot instrument (ProteinSimple) per manufacturer's instructions. Analysis was performed using Compass software (ProteinSimple).

### Phagocytic Activity Assay

Phagocytosis was measured with a kit (Cayman Chemical Company, Ann Arbor, MI). THP-1 cells or isolated human monocytes were suspended at a concentration of 1 × 10^6^ cells/ml in RPMI 1640 medium. 100 μl of cells were seeded into each well of a 96-well plate and incubated overnight with 1 μg/ml IgG1, isatuximab, Isa^*^, or 2 nM phorbol myristate acetate (PMA; positive control) and with latex beads-rabbit IgG-FITC complex added at a final dilution of 1:100. The next day, the cells in the plate were centrifuged at 400 *g* for 5 min and the supernatant was removed. Cells were resuspended in 200 μl of assay buffer and the fluorescent intensity was measured. Briefly, the average fluorescence intensity of a group of negative control (medium alone) was subtracted from positive control (PMA-treated) wells, yielding the net positive reading. This value represents phagocytosis under normal physiological conditions. The average fluorescence intensity of a group of negative control wells was subtracted from a group of identical experimental wells, yielding the net experimental reading, representing phagocytosis in response to the antibody. The percentage of phagocytic response to the antibody was determined as follows: % phagocytosis = net experimental reading / net positive reading × 100%. Phagocytic cells were also visualized by fluorescence microscopy with a Nikon Eclipse microscope (Tokyo, Japan) at 40× magnification. Uptake of IgG-FITC labeled beads was visualized directly in culture with no additional washing steps.

### CDC Assay

Approximately 75,000 cells in 50 μl cell culture medium were mixed with 25 μl of isatuximab or control human IgG1 diluted in culture medium (final concentration 0–10 μg/ml) and incubated on ice for 20 min. Human complement (25 μl at 20%, diluted from 100% with cell culture medium) was added to each well and the plate was incubated at 37°C (5% CO_2_) for 1 h. For assessing cell viability, 12 μl of alamarBlue was added to each well and incubation was continued for an additional 3 h. Resulting fluorescence signals were measured with an EnVision plate reader with excitation 560 nm and emission 590 nm. The CDC effect was calculated and presented as the percentage of cell viability: % cell viability = (test sample – blank control) / (cells with complement – blank control) ×100.

To inhibit CD59 on the cell surface, 75,000 test cells in 25 μl culture medium were pre-incubated with 25 μl of rat antihuman CD59 antibody or rat IgG2a isotype control antibody (140 μg/ml, final 3.5 μg antibody/test) on ice for 30 min before addition of isatuximab (final concentration 0–10 μg/ml), complement, and alamarBlue to measure CDC activity as described above.

### C3b Deposition

Approximately 150,000 test cells were incubated with or without isatuximab or control hIgG1 (final concentration 10 μg/ml) in a round-bottom 96-well plate on ice for 30 min. Human complement diluted with culture medium was added (final concentration 5%). Cells were incubated at 37°C (5% CO_2_) for 30 min, then washed twice with ice-cold PBS before incubation with the FITC-conjugated goat antihuman complement C3 antibody on ice for 30 min. After washing, C3 antibody binding to the cell surface was measured by flow cytometry using a FACSCalibur and analyzed using CellQuest Pro (v5.2).

## Results

### NK Cells and Monocytes Express Higher CD38 Levels Compared With T and B Cells

**Figure 1:**
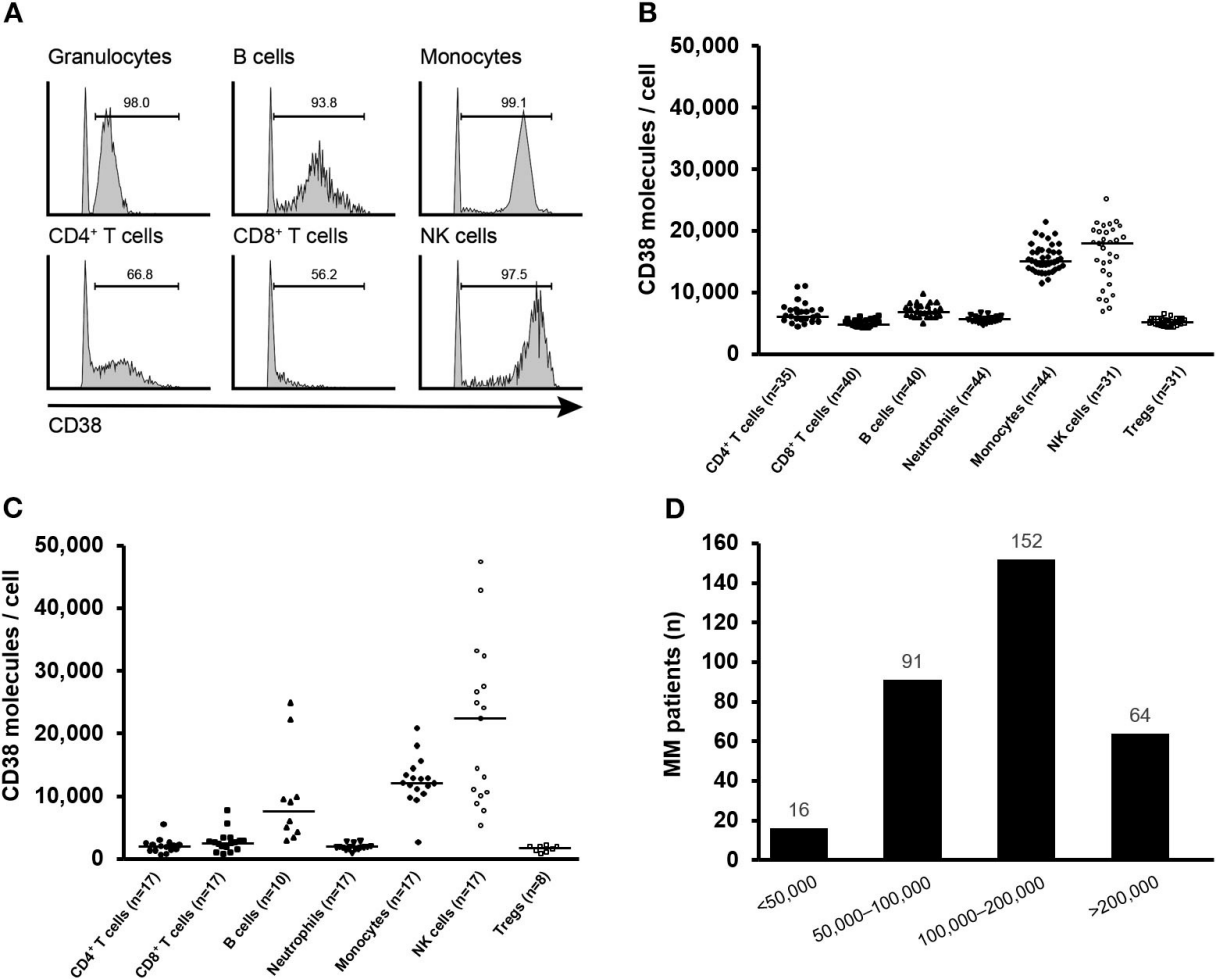
CD38 expression on human immune cells. **(A)** Representative histograms of CD38 expression on indicated immune cell populations of human PBMCs. Gates for CD38^+^ population were set relative to an isotype control. **(B)** Median CD38 RD of CD38^+^ CD4^+^ T cells, CD8^+^ T cells, B cells, neutrophils, monocytes, NK cells, and Tregs obtained from healthy donor PBMCs and **(C)** bone marrow from patients with MM. **(D)** CD38 receptor density distribution on myeloma cells from MM patients (*n* = 323). MM, multiple myeloma; NK, natural killer; PBMC, peripheral blood mononuclear cell; RD, receptor density.

We first analyzed CD38 expression in human PBMCs from healthy donors by flow cytometry. The gating strategy for detection of the major immune cell populations in PBMCs is illustrated in Supplementary Figure 1. CD38 was expressed on the surface of the tested immune cell populations, including CD4^+^ T cells, CD8^+^ T cells, B cells, NK cells, granulocytes/neutrophils, and monocytes. While more than 90% of NK cells, neutrophils, and monocytes were CD38^+^, the percentage of CD38^+^ lymphocytes was significantly lower (Figure 1A). In healthy donor PBMCs, NK cells and monocytes expressed a median >10,000 CD38 molecules/cell, whereas and the rest of immune cell lineages/subtypes expressed a median <4,000 CD38 molecules/cell (Figure 1B). To understand the CD38 expression in patients with MM, we analyzed immune cells from healthy donors and MM patients from an ongoing study (NCT04045795). Results for CD38 receptor density measurement demonstrated that the majority of immune cells from the bone marrow of patients with MM have similar levels of CD38 expression compared with peripheral immune cells from healthy donors with the exception of B cells and NK cells, which exhibit a trend of elevated CD38 receptor density (Figures 1B,C).

We further analyzed the expression of CD38 on myeloma cells from 323 MM patients enrolled in isatuximab clinical trials. Two-thirds of patients showed at least 10-fold higher CD38 receptor density than monocytes and NK cells from healthy individuals. Only 16 (5.0%) patients had CD38 expression <50,000 molecules/cell (Figure 1D). Collectively, the expression pattern of CD38 indicates that CD38 is tumor cell-associated antigen.

### Different Mechanisms Mediate Isatuximab-Induced Cytotoxicity Against MM Cells and DLBCL Cells

**Figure 2:**
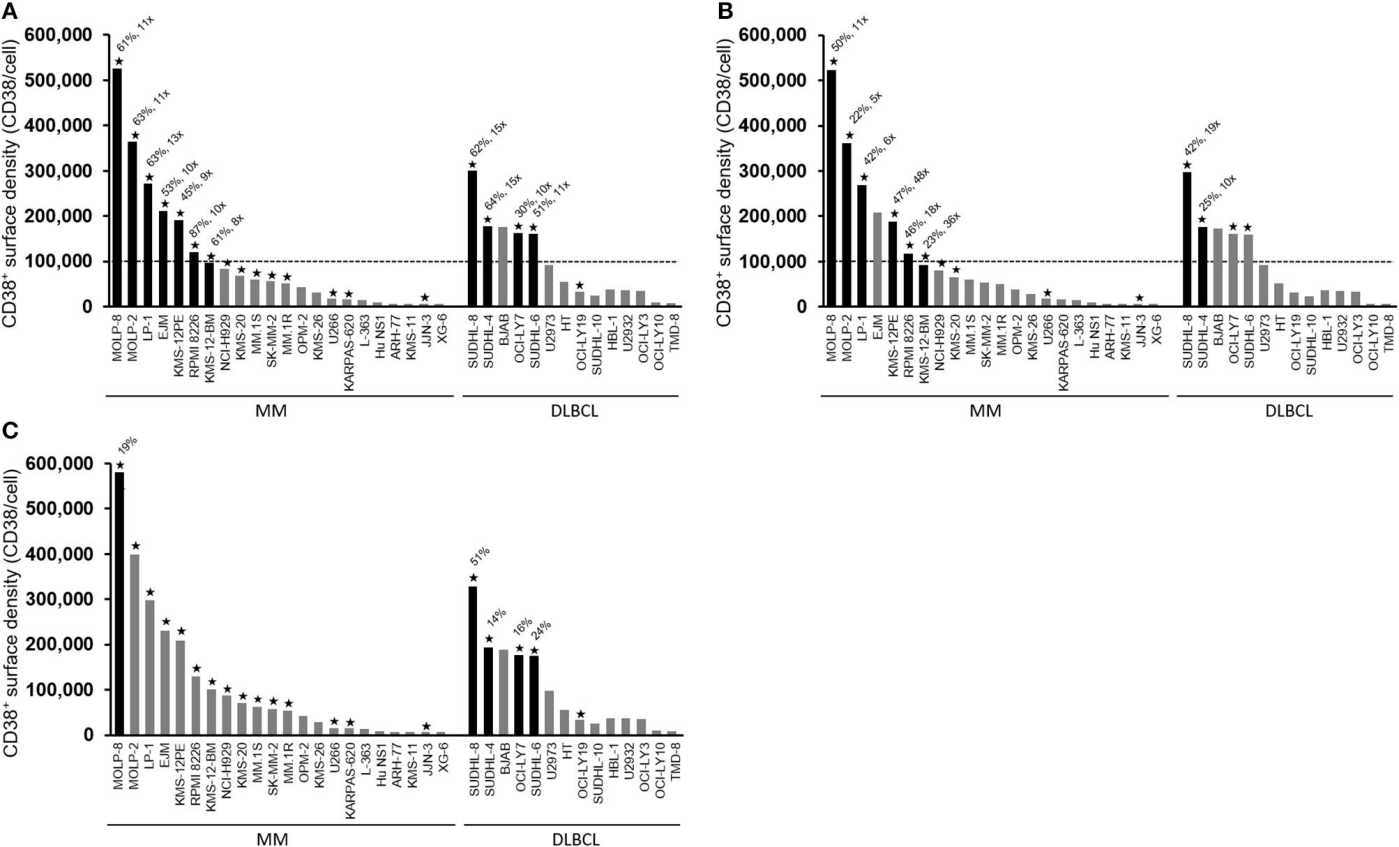
Isatuximab-mediated cytotoxic activities in MM and DLBCL cell lines. CD38 RD (y-axis) was measured in a panel of 22 MM and 14 DLBCL cell lines. Bars show CD38 RD. Data are representative of at least two independent experiments. **(A)** Cell lines marked with stars were tested for their sensitivity to isatuximab-induced ADCC lysis by NK-92.FcγRIIIA^V/V^ effector cells. Numbers on top of the bars represent the percentage of cell lysis by isatuximab-mediated ADCC and the fold change of cell lysis between isatuximab- and IgG1 isotype-mediated ADCC. Black solid bars indicate >30% target cell lysis and >2-fold increase in lysis during ADCC induction with isatuximab. The dotted line is the estimated threshold of CD38 RD required to trigger isatuximab-mediated ADCC. **(B)** Tumor cells and effector THP-1 cells were co-cultured in the presence of 1 μg/ml of isatuximab or isotype control hIgG1. Phagocytosis by effector THP-1 cells was quantified by flow cytometry. Numbers represent the percentage of phagocytosis in the presence of isatuximab and the fold increase in phagocytic activity induced by isatuximab compared with hIgG1-treated samples. Stars indicate the cell lines tested in the ADCP assay. Black solid bars represent tumor cells responsive to isatuximab-mediated ADCP, which is defined by >20% phagocytosis and a >2-fold increase in phagocytic activity with isatuximab- compared with hIgG1-treated samples. The dotted line indicates the suggested CD38 RD threshold of 100,000 molecules/cell. **(C)** Cells were treated with 10 μg/ml of isatuximab or hIgG1 to induce apoptosis. Stars indicate the cell lines used to evaluate isatuximab pro-apoptotic activity measured by annexin V staining. Black solid bars represent tumor cells where isatuximab treatment led to >10% apoptosis and >2-fold increased apoptosis compared with IgG treatment. ADCC, antibody-dependent cellular cytotoxicity; ADCP, antibody-dependent cellular phagocytosis; DLBCL, diffuse large B-cell lymphoma; (h)IgG1, (human) immunoglobulin G1; MM, multiple myeloma; RD, receptor density.

To better understand the mechanism of isatuximab-mediated cytotoxicity, we assessed the activity of different isatuximab-mediated cytotoxic pathways in 22 MM and 14 DLBCL cell lines in which we also quantified CD38 receptor density. Isatuximab-mediated ADCC was observed in 7/15 tested MM cell lines and 4/5 tested DLBCL cell lines. All the ADCC-sensitive cell lines displayed high CD38 receptor density (>100,000 molecules/cell) (Figure 2A). Similarly, these ADCC-sensitive MM cells, with one exception, were sensitive to isatuximab-mediated ADCP, while not all ADCC-sensitive DLBCL cells were responsive to isatuximab-mediated ADCP (Figure 2B). When isatuximab-mediated CDC was analyzed among the same MM and DLBCL cell lines, only one MM cell line, LP-1, and one DLBCL cell line, SUDHL-8, were susceptible to isatuximab-triggered cell lysis (data not shown). Both these cell lines had a high CD38 receptor density (>250,000 CD38 molecules/cell). In addition, of all the MM cell lines tested for isatuximab-induced apoptosis, only MOLP-8 cells were sensitive. Interestingly, the DLBCL cells were more sensitive than MM cells to isatuximab-induced apoptosis (Figure 2C). Taken together, these data indicate that ADCC and ADCP are the most prevalent isatuximab-mediated mechanisms of cell death in MM cells, whereas ADCC and apoptosis appear to be the most common routes in DLBCL cells.

### CD38 Receptor Density Impacts Isatuximab-Mediated ADCC

**Figure 3:**
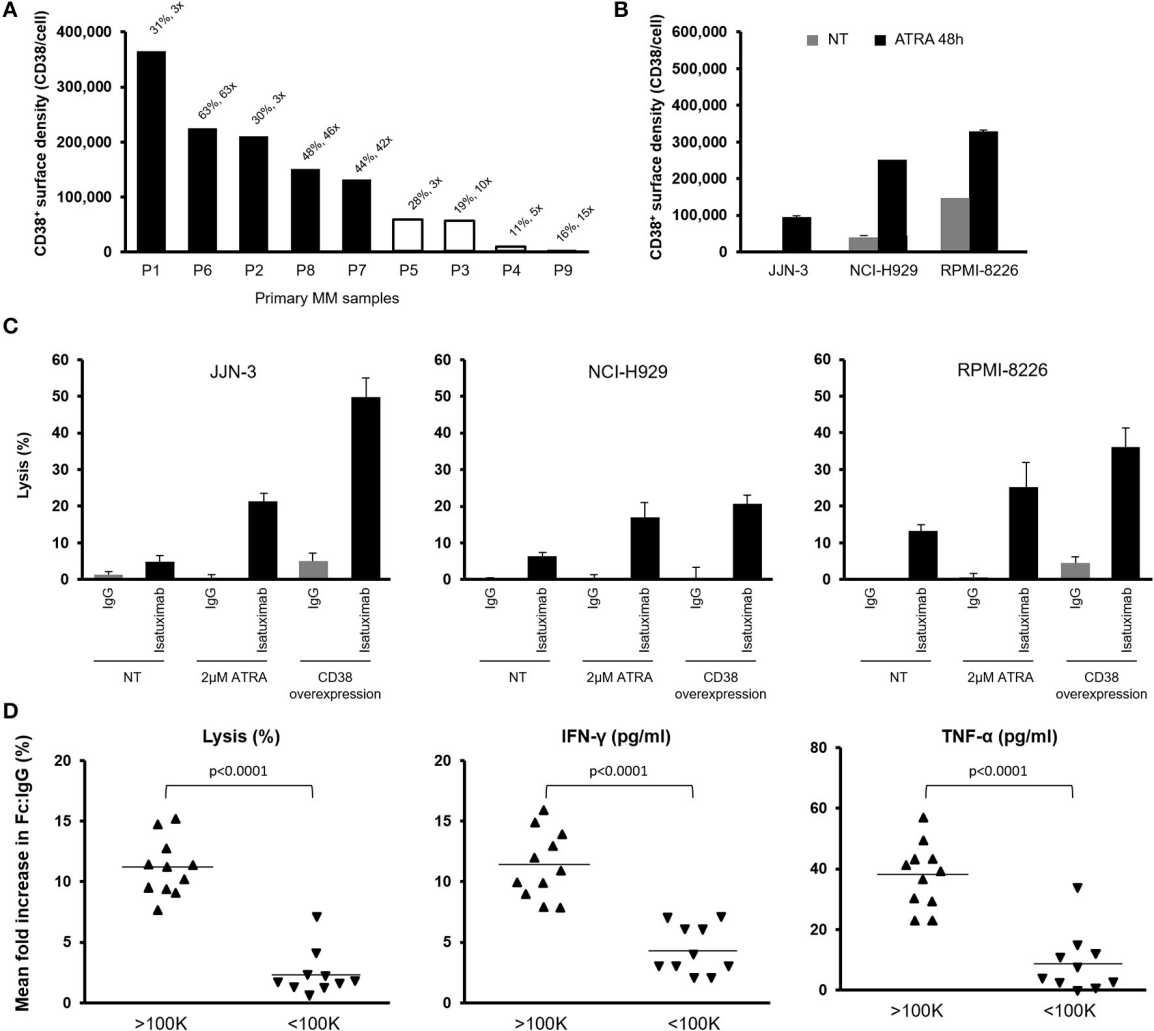
Effect of CD38 RD on isatuximab-induced ADCC against primary MM cells. **(A)** Nine primary MM patient tumor cells were tested for their sensitivity to isatuximab-induced ADCC. The percentage of ADCC lysis was measured by calcein AM release from the tumor cells and the fold change relative to the IgG1 isotype control-treated samples expressed on top of the bars. Black solid bars indicate >30% lysis and a >2-fold increase in cell lysis after treatment with isatuximab vs. IgG1 isotype control. **(B)** MM cells were treated with 2 μM of ATRA for 48 h. Mean (SD) CD38 RD was quantified for cells with (black bars) or without (gray bars) ATRA treatment (*n* = 3). **(C)** Indicated MM cells were pretreated with or without 2 μM ATRA for 48 h and mixed with NK-92.CD16^V/V^ effector cells in the presence of 1 μg/ml isatuximab or IgG1 isotype control. Mean (SD) NK cell-mediated cytolytic activities were measured by calcein AM release (*n* = 3). Cells overexpressing CD38 were used as positive controls of isatuximab-mediated ADCC. **(D)** 16 MM and 5 DLBCL cell lines were treated with isatuximab or IgG1 isotype control (1 μg/ml) for 5 h. Mean fold increase in the percentage of target cell lysis, IFN-γ production, and TNF-α production is shown for target cells grouped by CD38 expression level, either above or below the estimated threshold for ADCC (1 × 10^5^ molecules/cell; *n* = 1 for each cell line). Significance assessed by two-tailed *t*-test. ADCC, antibody-dependent cellular cytotoxicity; ATRA, all-trans retinoic acid; DLBCL, diffuse large B-cell lymphoma; IFN-γ, interferon-gamma; IgG1, immunoglobulin G1; MM, multiple myeloma; NK, natural killer; RD, receptor density; SD, standard deviation; TNF-α, tumor necrosis factor-alpha.

To confirm the observations made in MM cell lines, we tested isatuximab-mediated ADCC in primary myeloma cells isolated from MM patients. Results showed isatuximab-mediated ADCC in five out of nine primary myeloma samples. Susceptibility to ADCC was associated with CD38 receptor density, requiring >100,000 molecules/cell (Figure 3A).

To test the association between CD38 expression and isatuximab-mediated ADCC, selected MM cell lines (JJN-3, NCI-H929, and RPMI-8226) were treated for 48 h with ATRA, a potent CD38 inducer (34, 35), thus increasing CD38 expression (>100,000 molecules/cell) (Figure 3B). Cells pretreated with ATRA became more sensitive to isatuximab-mediated ADCC. At the same time, we tested ADCC activity in the MM cell lines engineered to overexpress CD38. Isatuximab-mediated ADCC was higher in the CD38-overexpressing cells compared with parental cells, with activity comparable to ATRA-treated MM cells (Figure 3C). Therefore, enhancing CD38 expression level on target tumor cells with ATRA treatment seems to contribute to improved isatuximab-induced ADCC of MM cells.

Interestingly, the expression level of CD38 on tumor cells also affected NK cell activation in isatuximab-mediated ADCC. Higher levels of IFN-γ and TNF-α production by NK cells, and greater cell lysis, were found for the tumor cells with higher CD38 receptor density (>100,000 molecules/cell vs. <100,000 molecules/cell) (Figure 3D). Our observations suggest that efficient isatuximab-mediated killing among high CD38-expressing MM and DLBCL cells is at least partially related to more robust NK cell activation. Therefore, factors that can affect NK cell functionality would likely have a critical impact on isatuximab-mediated killing of target tumor cells.

We further investigated the impact of isatuximab on CD38^+^, CD34^+^ bone marrow cells from healthy donors. The CD38 receptor density was comparable with that on NK cells and monocytes in normal peripheral blood but was significantly lower than that on MM cells. We also found that isatuximab exposure resulted in less CD34^+^ cell lysis in the ADCC analysis. In progenitor cell assays, when the cells were treated isatuximab or isotype control during *in vitro* differentiation, we did not detect isatuximab affecting progenitor cell capacity measured by burst-forming unit-erythroid (BFU-E), colony-forming unit-granulocyte macrophage (CFU-GM), and colony-forming unit megakaryocyte (CFU-Meg) (Supplementary Figure 2). Taken together, the results indicate that isatuximab has preferential cytotoxic effect toward high CD38-expressing tumor cells but spares low CD38-expressing cells in normal tissues.

### MM Cells Suppress Isatuximab-Mediated ADCC via the PD-L1/PD-1 Pathway

**Figure 4:**
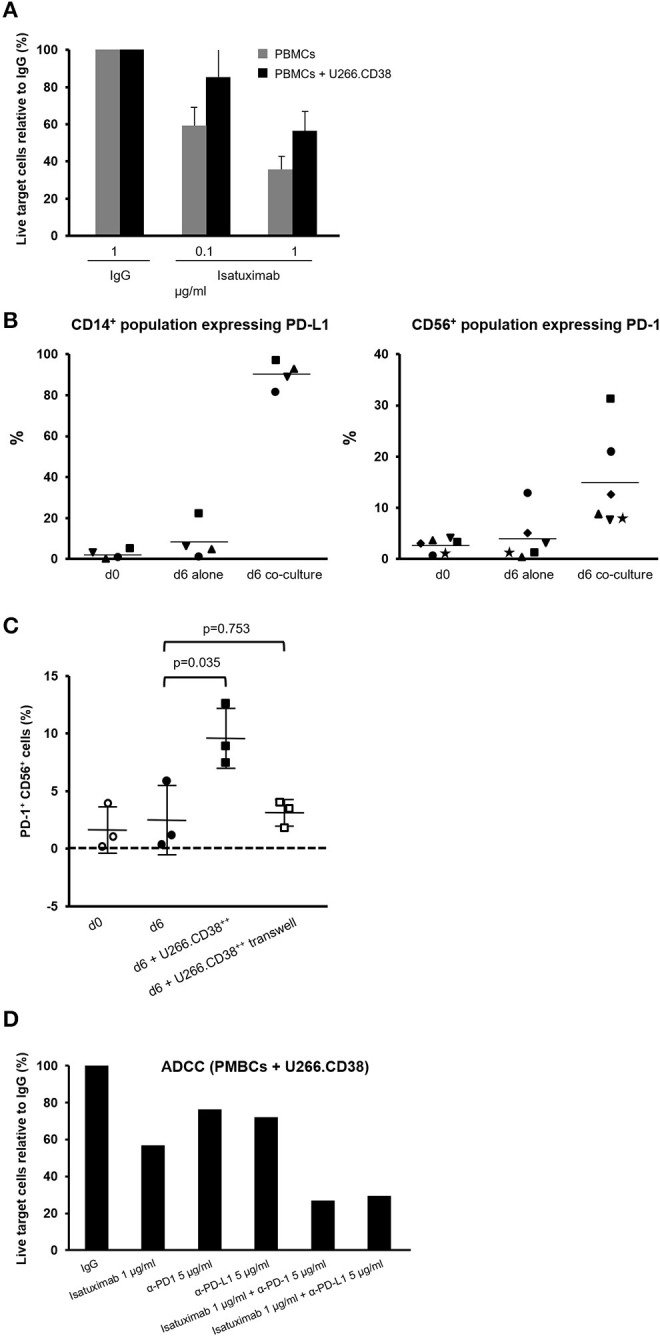
Effect of PD-L1/PD-1 interaction on isatuximab ADCC activity. **(A)** 5 × 10^6^ PBMCs and 5 × 10^5^ U266 cells overexpressing CD38 (U266.CD38^++^) were co-cultured in ultra-low attachment six-well plates for 7 days. The pre-conditioned PBMCs were then mixed with fresh U266.CD38^++^ cells with the presence of indicated concentrations of isatuximab or IgG1 isotype control at 37°C for 2–3 h to induce ADCC. Live CD138^+^ cells were quantified by flow cytometry. The percentages of live target cells were calculated relative to those for the IgG1 control. Each treatment was analyzed in triplicate. Results are Mean (SD). **(B)** PBMCs were pre-conditioned by MM cells as described in **(A)**. The cells were analyzed for expression of PD-1 on CD56^+^ cells (NK cells) and PD-L1 on CD14^+^ cells (monocytes) by flow cytometry on day 0 and day 6 of the co-culture (*n* = 4–6). **(C)** 5 × 10^6^ PBMCs and 5 × 10^5^ U266 cells overexpressing CD38 (U266.CD38^++^) were co-cultured in ultra-low attachment six-well plates or a transwell system to prevent direct contact between MM cells and PBMCs for 6 days. The cells were analyzed for expression of PD-1 on CD56^+^ cells (NK cells) by flow cytometry on day 0 and day 6 of the co-culture. **(D)** PBMCs were cultured with or without U266.CD38^++^ cells for 6 days as described in **(A)**. The cells were subsequently mixed with fresh U266.CD38^++^ target cells in the presence of 1 μg/ml of isatuximab or IgG1 isotype control at 37°C for 2–3 h. For the cells that received co-treatment, 5 μg/ml of anti-PD-1 or anti-PD-L1 antibody was added 30 min before adding 1 μg/ml of isatuximab to trigger ADCC. Following isatuximab treatment, live CD138^+^ cells (U266.CD38^++^ cells) were quantified by flow cytometry. ADCC, antibody-dependent cellular cytotoxicity; IFN-γ, interferon-gamma; IgG1, immunoglobulin G1; IL-6, interleukin-6; MM, multiple myeloma; NK, natural killer; NT, not treated; PBMC, peripheral blood mononuclear cell; PD-1, programmed cell death-1; PD-L1, programmed cell death-ligand 1; PD-L2, programmed cell death-ligand 2; SD, standard deviation; TGF-β, transforming growth factor-beta.

To test whether MM cells can suppress isatuximab-mediated ADCC, we co-cultured U266.CD38^++^ cells (U266 cells stably overexpressing CD38) with PBMCs from healthy donors for 7 days. Following the 7-day co-culture, we added isatuximab and freshly prepared U266.CD38^++^ target cells to measure isatuximab-induced ADCC. The experiment revealed that co-culture of PBMCs and MM cells resulted in attenuated lysis of the target cells compared with isatuximab-induced killing by PBMCs from the same donor without prior MM cell co-culture (Figure 4A).

We analyzed PD-1 and PD-L1 expression on PBMCs at different time points during their co-culture with MM cells, to investigate whether this could contribute to the suppression of NK cell function. PD-1 expression was detectable on NK cells as early as 1 day after co-culture and increased up to day 8. Although there was evidence for induction of PD-1 expression in B cells and T cells, PD-1 was most strongly induced in NK cells in the presence of MM cells (Supplementary Figure 3). The induction of PD-L1 expression was predominantly found in monocytes. Almost all of the monocytes expressed PD-L1, detectable on about 50% of monocytes on day 1 of the co-culture, increasing to almost 100% by day 8 (Supplementary Figure 3). To confirm these findings, we analyzed PD-1 and PD-L1 expression on PBMCs from up to six healthy donors following a 6-day co-culture with MM cells. Results consistently showed PD-L1 induction on monocytes (the mean proportion of PD-L1^+^ monocytes was 2, 8, and 91% on day 0, day 6, and day 6 co-culture) and PD-1 expression on NK cells on day 6 of the co-culture (the mean proportion of PD-1^+^ NK cells was 3, 4, and 15% on day 0, day 6, and day 6 co-culture, respectively) (Figure 4B). We performed a further transwell experiment by separating U266.CD38^++^ cells and PBMCs during cell culture. We did not find increased PD-1 expression (Figure 4C), indicating that the direct contact between tumor cells and NK cells, but not soluble signal molecules such as cytokines, is essential for PD-1 induction in NK cells.

To confirm that the PD-L1/PD-1 pathway suppresses isatuximab-mediated ADCC, we co-cultured U266.CD38^++^ cells and healthy donor PBMCs for 7 days and performed ADCC experiments in the presence of anti-PD-1 or anti-PD-L1 antibodies. Isatuximab alone at a concentration of 1 μg/ml killed slightly more than 40% of the co-cultured MM target cells. However, when isatuximab was combined with either anti-PD-1 or anti-PD-L1 antibody, the combined treatment induced killing of 70–80% of the target cells, indicating that PD-1 blockade synergistically enhanced isatuximab-mediated ADCC (Figure 4D). These data suggest that MM cells in co-culture with PMBCs can induce the expression of PD-1 and PD-L1, suppressing NK cell function and thereby compromising isatuximab-induced cytotoxicity.

In addition, we quantified expression of PD-L1 and PD-L2 in MM cell lines, and investigated whether cytokines present in the MM tumor environment, such as IL-6 and TGF-β1, could influence expression levels. Similar to the positive control IFN-γ, IL-6 strongly induced PD-L1 expression on NCI-H929, RPMI-8226, and U266 cells, while TGF-β1 did not. PD-L2 expression was not induced by cytokine stimulation (Supplementary Figure 4). The expression of PD-L1 on MM cell lines suggests that, like PD-L1^+^ monocytes, MM cells could also suppress NK cell function via the PD-L1/PD-1 pathway. The further induction of PD-L1 with cytokine stimulation suggests that in the bone marrow environment in MM patients, PD-L1 expression on MM cells could contribute to escape from antitumor immunity.

### TGF-β1 Released by MM Cells Can Suppress Isatuximab-Mediated ADCC

**Figure 5:**
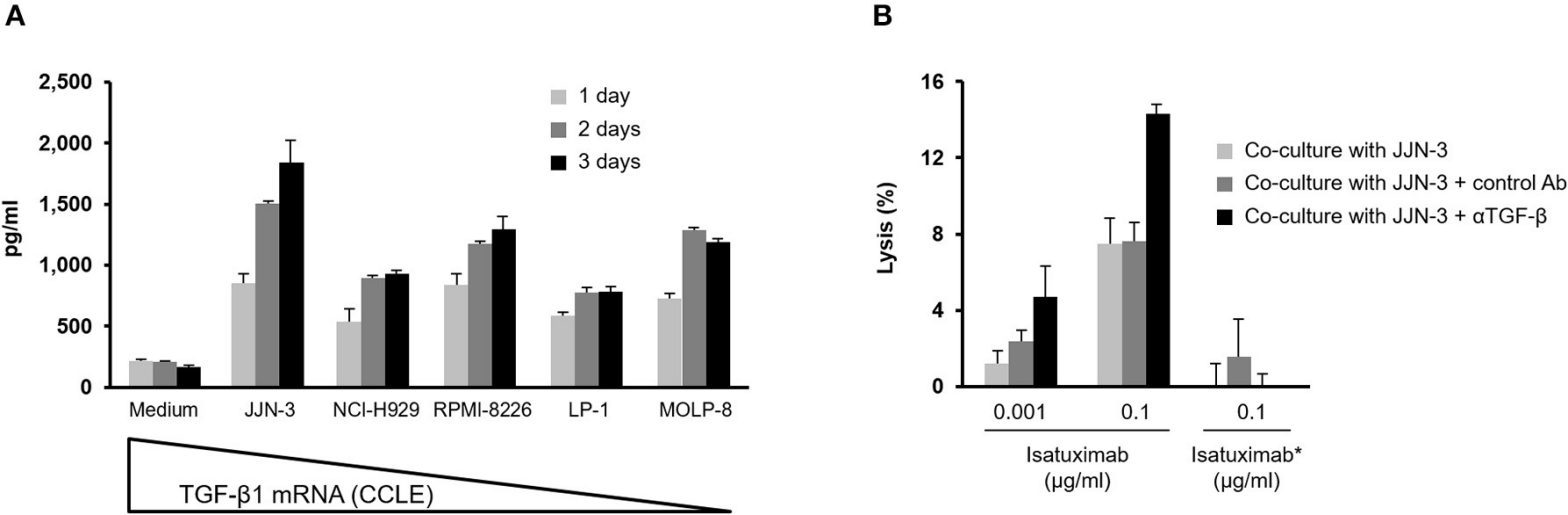
Effect of TGF-β on isatuximab-induced ADCC activity. **(A)** 1 × 10^6^ MM cells were cultured in 100 μl of complete medium. Culture medium supernatant was collected on days 1, 2, and 3; TGF-β1 secretion was determined by MSD Human TGF-β1 Kit. TGF-β mRNA expression data from CCLE. **(B)** TGF-β neutralization, using an anti-TGF-β1,2,3 antibody, in JJN-3 tumor cell supernatants restored isatuximab-induced ADCC. A transwell system was used to co-culture 2 × 10^6^ purified NK cells with 6 × 10^6^ JJN-3 cells in the presence of 50 μg/ml of anti-TGF-β antibody or isotype control at 37°C for 90 h. To quantify isatuximab-triggered ADCC, 4 × 10^4^ calcein AM-labeled RPMI-8226 cells were mixed with 1.2 × 10^5^ NK cells in the presence of isatuximab or Isa* at indicated concentrations and calcein AM release quantified. Analyses were performed in triplicate. Results are mean (SD). Ab, antibody; ADCC, antibody-dependent cellular cytotoxicity; CCLE, Cancer Cell Line Encyclopedia; Isa*, isatuximab mutant unable to bind to CD38; mRNA, messenger ribonucleic acid; MSD, Meso Scale Diagnostics; NK, natural killer; SD, standard deviation; TGF-β1, transforming growth factor-beta 1.

Given the immunosuppressive role of TGF-β1, we investigated whether it could negatively impact isatuximab-induced ADCC by treating NK-92 cells expressing the high-affinity variant of CD16, NK-92.CD16^V/V^ cells, with recombinant TGF-β1 prior to measuring ADCC activity. Following 90 h' pre-conditioning with 10 ng/ml recombinant TGF-β1, both the cytolytic activity and isatuximab-mediated ADCC against target MM cells were decreased (Supplementary Figure 5). Significant levels of TGF-β1 were secreted by MM cell lines cultured *in vitro* for up to 3 days (Figure 5A). We co-cultured TGF-β1-producing JJN-3 cells with purified primary NK cells, using a transwell system to prevent direct contact between MM cells and primary NK cells, in the presence of either anti-TGF-β1 or an isotype control antibody. After 4 days of co-culture, the ability of the pre-conditioned PBMCs to carry out isatuximab-mediated ADCC was tested, with the CD38-expressing RPMI-8226 cells used as the target. Results indicated that the TGF-β1 neutralizing antibody enhanced isatuximab-mediated ADCC of the target cells by the pre-conditioned PBMCs (Figure 5B). Taken together, this demonstrates that TGF-β1 secreted by MM cells suppresses NK cell cytolytic activity, which could potentially compromise isatuximab-mediated killing of CD38-expressing MM cells.

### Isatuximab Directly Activates NK Cells via Crosslinking CD16 and CD38

**Figure 6:**
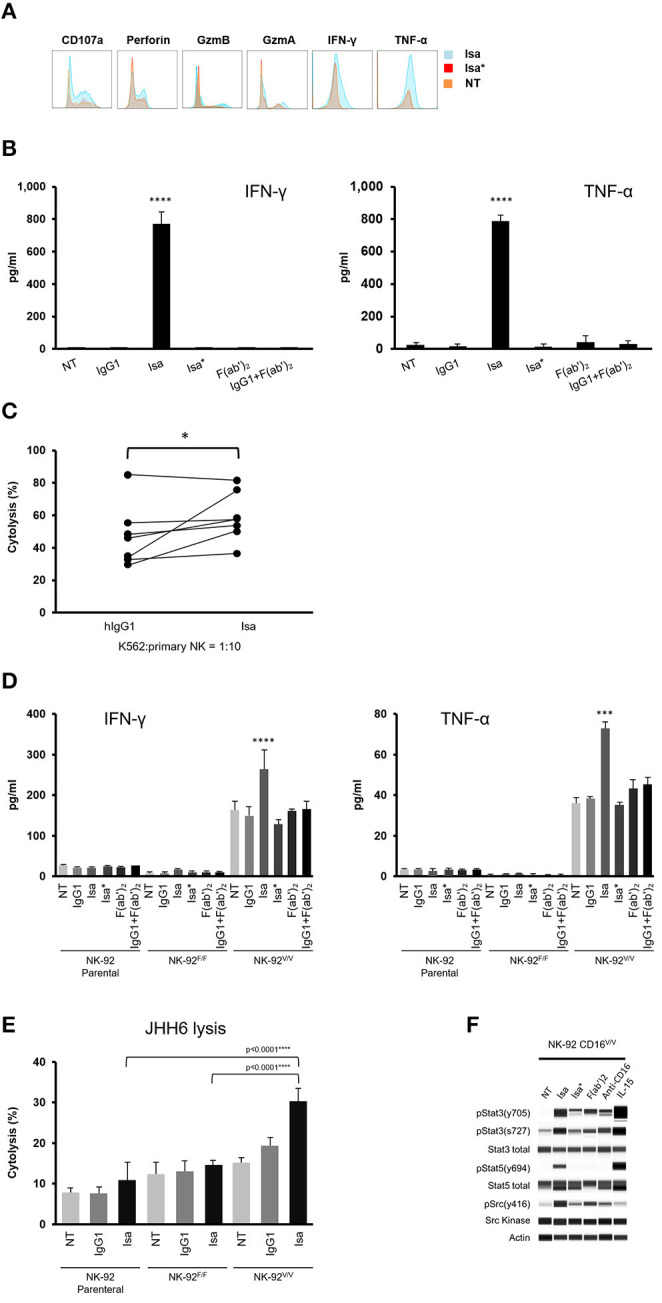
Isatuximab activates NK cells by crosslinking CD38 and CD16. **(A)** PBMCs were treated with or without 1 μg/ml of Isa* or Isa. After 7 days, NK cell activity was analyzed by intracellular staining for the production of perforin, granzyme-A, granzyme-B, IFN-γ, and TNF-α. **(B)** 1 × 10^6^ cells/ml of purified human NK cells (*n* = 3) were cultured overnight with or without the presence of IgG1, Isa, Isa*, F(ab')_2_, and IgG1+F(ab')_2_. Cell culture supernatants were collected and analyzed for the production of IFN-γ and TNF-α by MSD. ****p < 0.001 by unpaired *t*-test (*n* = 3). **(C)** K562 cells were labeled with calcein AM and co-cultured with purified human NK cells (*n* = 7) at the ratio 1:10 in the presence of 1 μg/ml of IgG1 or Isa for 1 h and cytotoxicity measured by calcein AM release. The experiment was performed in technical triplicate, with statistical analysis by two-tailed paired *t*-test. *p < 0.05. **(D)** 1 × 10^6^ cells/ml of human NK-92 parental, NK-92.CD16^F/F^ or NK92.CD16^V/V^ cells (*n* = 3) were cultured overnight with or without the presence of 1 μg/ml of IgG1, Isa, Isa*, F(ab')_2_, or IgG1+F(ab')_2_. Culture supernatants were collected and assayed for the production of IFN-γ and TNF-α by MSD. ***p < 0.001 by unpaired *t*-test (*n* = 3). **(E)** Cytotoxicity against CD38^−^ JHH6 cells (*n* = 3) was measured by calcein AM release. The experiment was conducted in technical triplicate. ****p < 0.0001 by unpaired *t*-test (*n* = 3). **(F)** NK92.CD16^V/V^ cells were serum-starved overnight and treated with or without Isa, Isa*, F(ab')_2_, anti-CD16, IL-15 for 15 min prior to cell lysis. Western blotting was used to determine activation of the Src kinase, STAT3 and STAT5. All results are mean (SD). F(ab')_2_, F(ab')_2_ portion of isatuximab; GzmA, granzyme-A; GzmB, granzyme-B; IFN-γ, interferon-gamma; IgG1, immunoglobulin G1; Isa, isatuximab; Isa*, isatuximab mutant unable to bind to CD38; MSD, Meso Scale Diagnostics; NK, natural killer; NT, not treated; PBMC, peripheral blood mononuclear cell; TNF-α, tumor necrosis factor-alpha.

Although at relatively low levels, CD38 is ubiquitously expressed on human immune cells. Monocytes and NK cells have the highest expression levels of CD38 among the immune cells in human peripheral blood (Figures 1A–C). Since CD38 interacts with CD16 in NK cell activation (27, 28), and as we hypothesized that isatuximab allows a cross-link between CD38 and CD16, we investigated its functional impact on NK cells. Therefore, we treated PMBCs with isatuximab, and found that NK cells from isatuximab-treated PBMCs secreted a significant amount of perforin, granzymes, IFN-γ, and TNF-α. In addition, CD107a expression was increased. In contrast, NK cells from PBMCs that were treated with a mutant isatuximab, Isa^*^ (with mutations Y101K and Y102E on isatuximab to eliminate its ability to bind to CD38), had no such effect (Figure 6A). This indicates that CD16 stimulation alone by the Fc portion of isatuximab does not cause isatuximab-mediated NK cell stimulation in PBMCs.

To better characterize isatuximab-mediated NK cell activation, we treated NK cells purified from PBMCs with isatuximab, IgG1, Isa^*^, F(ab')_2_, or combined IgG1 and F(ab')_2_ for 24 h and then measured cytokines released into the culture media. Interestingly, only isatuximab triggered the release of IFN-γ and TNF-α. Neither engaging CD16 via IgG1 or Isa^*^ nor binding CD38 via F(ab')_2_ alone was sufficient to trigger robust cytokine release. Importantly, the combination of IgG1 and F(ab')_2_ also failed to do so, suggesting that crosslinking of CD16 and CD38, rather than purely simultaneous engagement, is necessary to drive such robust TNF-α and IFN-γ release (Figure 6B). We then sought to test whether isatuximab-activated NK cells were able to efficiently lyse CD38^−^ tumor cells. For this, NK cells purified from PBMCs were used in a cytotoxicity assay against CD38^−^ K562 cells. The lysis of K562 cells by isatuximab-treated NK cells obtained from seven healthy donors was higher than the lysis by IgG1-treated NK cells in all but one case (Figure 6C). These data indicate that, in addition to its role in NK-mediated ADCC, isatuximab can also directly activate NK cells and increase their lytic activity against CD38^−^ tumor cells.

To study the signaling event during isatuximab-mediated NK cell activation, we decided to use the human CD38-expressing NK cell line, NK-92, that does not express CD16 (36), alongside two cell lines stably expressing either low-affinity variant 158F.CD16 (NK-92^F/F^) or high-affinity variant 158V.CD16 (NK-92^V/V^). Cell lines were treated with isatuximab, IgG1, Isa^*^, F(ab')_2_, or IgG1+F(ab')_2_ for 5 h, after which the cell culture supernatants were collected and analyzed for IFN-γ and TNF-α. Results indicated that supernatants from isatuximab-treated NK-92^V/V^ cells showed higher levels of IFN-γ and TNF-α production compared with cells that were non-treated or treated with IgG1, Isa^*^, F(ab')_2_, or IgG1+F(ab')_2_ (Figure 6D). Notably, non-treated NK-92^V/V^ cells produced high basal levels of IFN-γ and TNF-α compared with primary NK cells (Figure 6D vs. Figure 6B). Nonetheless, NK-92^V/V^ cells respond to isatuximab treatment similarly to primary NK cells with increased IFN-γ and TNF-α production (Figure 6D vs. Figure 6B). Furthermore, isatuximab-treated NK92^V/V^ cells showed significantly increased cytolytic activity against CD38^−^ liver cancer JHH6 cells when compared with IgG1-treated NK cells (Figure 6E). This result mimics the stimulatory effect of isatuximab on primary NK cells to enhance the cytolytic activity again CD38^−^ K562 cells (Figure 6C).

To understand how isatuximab regulates the signaling events in NK cells, we treated NK-92^V/V^ cells with Isa, Isa^*^, F(ab')_2_, agonist anti-CD16 antibody, and recombinant IL-15, respectively, for 15 min and then harvested the whole-cell lysates to analyze Src kinase activity. Isa^*^ and anti-CD16 antibody triggered similar levels of Src Y416 phosphorylation due to activation of CD16 signaling. F(ab')_2_ induced stronger phosphorylation of Src Y416, whereas a much more robust Src Y416 phosphorylation was detected in the samples stimulated by isatuximab. This result suggests that isatuximab crosslinks CD16 and CD38 to synergistically activate CD16 signaling. We did not find evidence of Src Y416 phosphorylation with IL-15 stimulation (Figure 6F). Interestingly, isatuximab stimulation also induced phosphorylation of both STAT3 and STAT5, similar to the effect of IL-15 simulation on NK-92^V/V^ cells. This result indicated that CD16 is probably not the only NK cell receptor with which CD38 interacts.

### Isatuximab Increases the Phagocytic Activity of Human Monocytes

**Figure 7:**
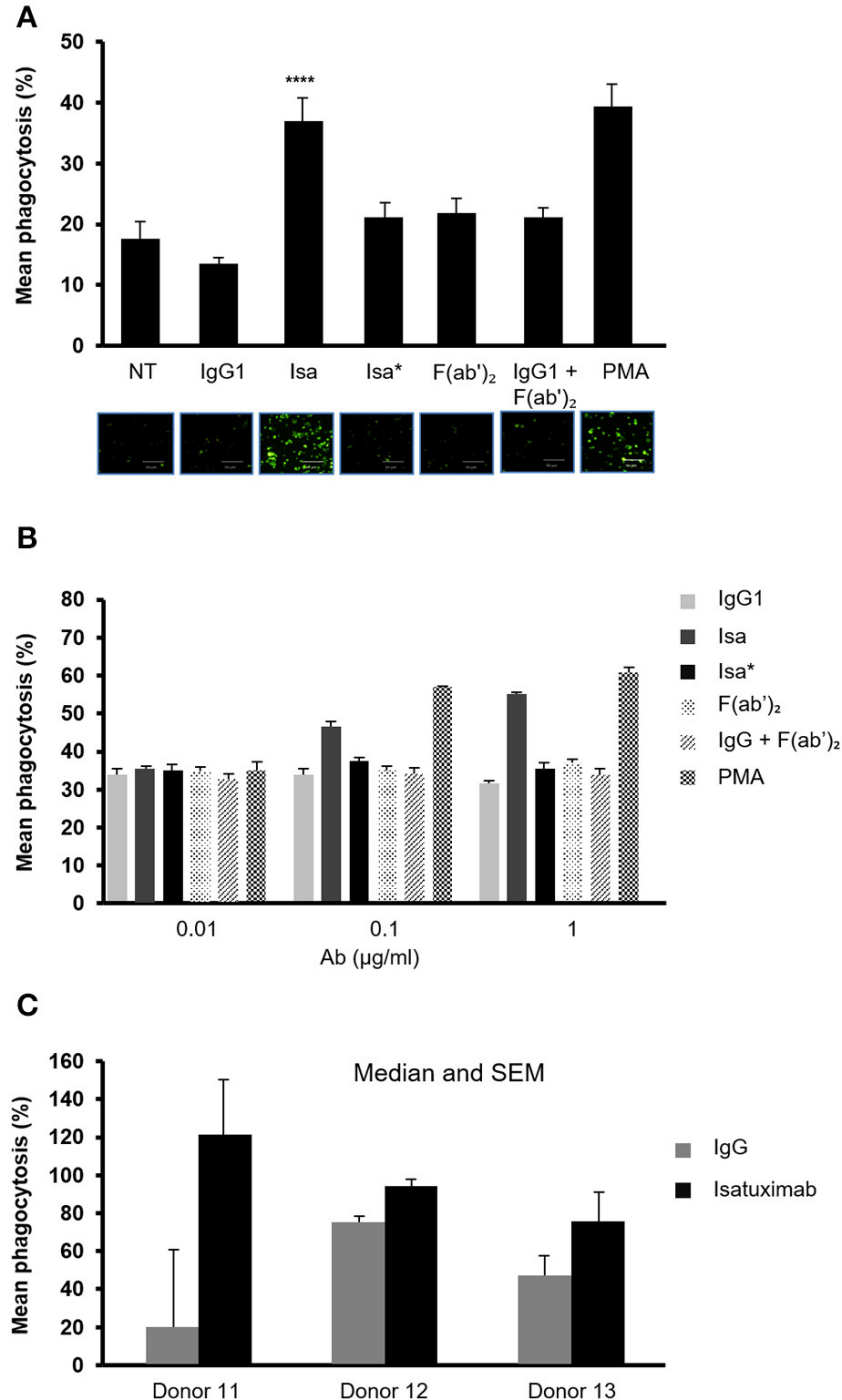
Effect of isatuximab on phagocytic activity of human monocytes **(A)** Human monocytic THP-1 cells (1 × 10^6^ cells/ml) were seeded overnight along with FITC-labeled latex-IgG beads in the presence or absence of 1 μg/ml of IgG1, Isa, Isa*, F(ab')_2_, IgG1+F(ab')_2_, or 2 μM of PMA. Fluorescence microscopic analysis of phagocytosis was followed by incubation of THP-1 cells with latex beads and antibody treatment to determine phagocytic activity (*n* = 3). ****p < 0.0001 by unpaired *t*-test (*n* = 3). **(B)** The phagocytosis assay described in **(A)** was repeated using titrations of the indicated treatments (*n* = 3). **(C)** Monocytes isolated from three healthy human donors were seeded overnight along with FITC-labeled latex-IgG beads in the presence of 1 μg/ml of IgG1 or Isa. The experiment was conducted in technical triplicate. Error bars are SD. Statistical tests performed using one-way ANOVA. F(ab')_2_, F(ab')_2_ portion of isatuximab; FITC, fluorescein isothiocyanate; IgG1, immunoglobulin G1; Isa, isatuximab; Isa*, isatuximab mutant unable to bind to CD38; PMA, phorbol myristate acetate.

Given that monocytes express CD38, FCγIIa/CD32, and FcγIIIa, we next investigated the ability of isatuximab to induce monocyte activation. For this purpose, we seeded THP-1 human monocytic cells and treated them overnight with isatuximab, IgG1, Isa^*^, F(ab')_2_, IgG1+F(ab')_2_, or PMA (as a positive control), along with latex beads conjugated with rabbit IgG-FITC. The fluorescent intensity of THP-1 cells was measured by flow cytometry to calculate the extent of phagocytosis of the fluorescent beads. Isatuximab significantly increased the THP-1 cell phagocytic activity, from 15 to 40%—a level similar to that of the PMA control (Figure 7A). This was also visualized by fluorescence microscopy (Figure 7A). There was a dose-dependent increase in phagocytosis with increasing concentrations of isatuximab and PMA, but no change with IgG1, Isa^*^, F(ab')_2_, or IgG1+F(ab')_2_ (Figure 7B). The ability of isatuximab to increase monocyte phagocytic activity, compared with IgG1, was confirmed in monocytes purified from the peripheral blood of healthy volunteers (Figure 7C). Taken together, this indicates that the isatuximab-mediated increase in phagocytosis requires both the F(ab')_2_ and Fc portions of the antibody, similar to our observations for NK cell activation.

### Isatuximab Induces C3b Deposition in Cell Lines With High CD38 Expression

Although two MM (LP-1) and DLBCL (SUDHL-8) cell lines with high CD38 receptor density (>250,000 molecules/cell) were susceptible to isatuximab-mediated CDC, other MM cell lines with a high CD38 receptor density—MOLP-8 and MOLP-2—were not, suggesting that high CD38 receptor density is insufficient for CDC to be induced by isatuximab.

**Figure 8:**
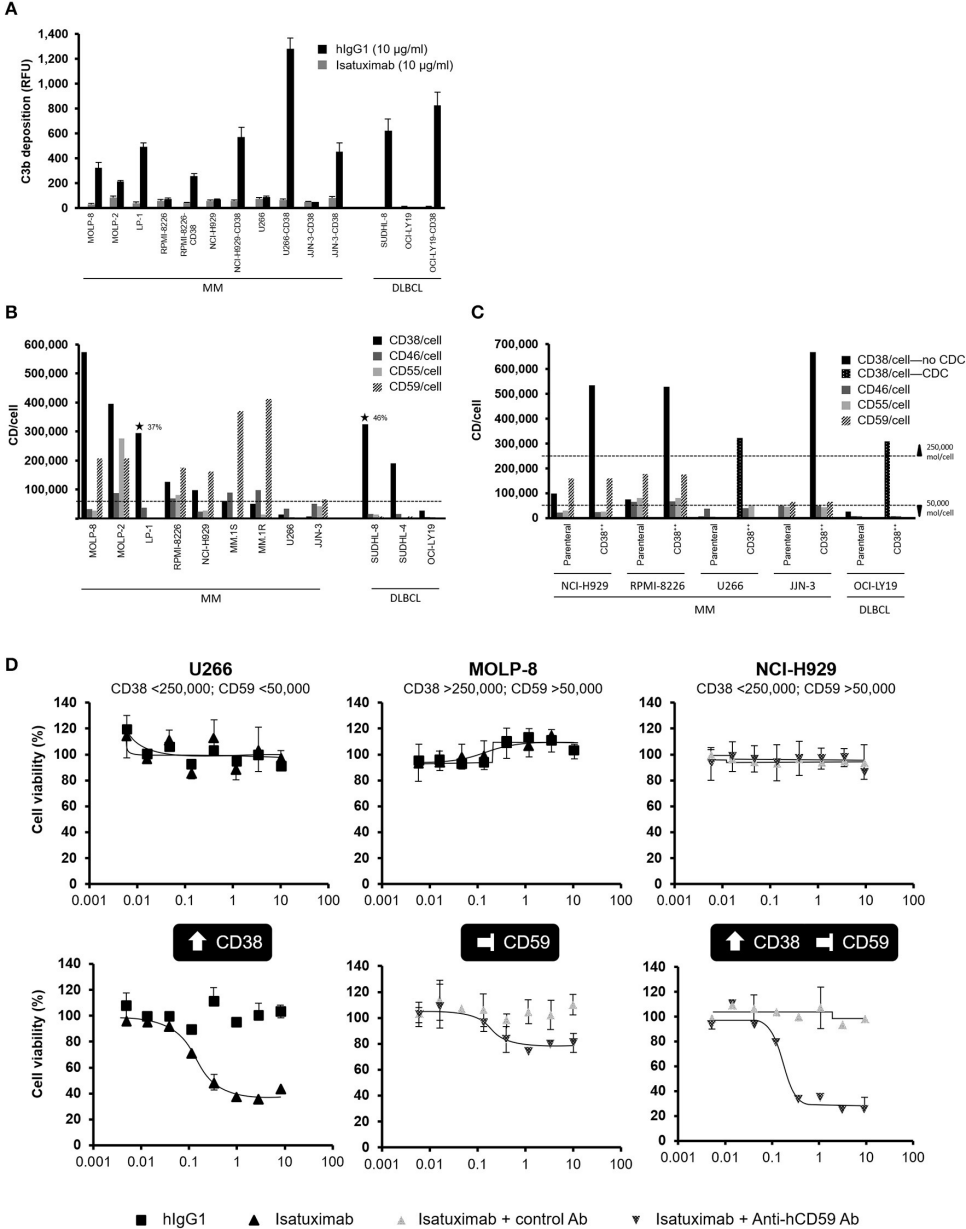
Expression of CD38 and complement regulatory proteins impact susceptibility to isatuximab-induced CDC. **(A)** C3b deposition was quantified by flow cytometry on the surface of MM and DLBCL cell lines, either with natural CD38 RD >250,000 molecules/cell, or for cell lines with parental cell line CD38 RD <250,000 molecules/cell with and without engineering to overexpress CD38 above this threshold. ⋆ indicates CDC sensitive cell lines. **(B)** Cell surface expression levels of CD38 and the complement regulatory proteins CD46, CD55, and CD59 were determined for MM and DLBCL cell lines by flow cytometry. **(C)** Complement regulatory protein expression was not affected by overexpression of CD38 in MM and DLBCL cell lines resistant to isatuximab-mediated CDC, for parental cell lines expressing high levels of complement regulatory proteins. **(D)** Isatuximab-triggered CDC lysis of resistant MM cell lines required increased CD38 expression (>250,000 molecules/cell) and functional inhibition of complement regulatory proteins such as CD59. Experiments were repeated at least twice, with each treatment analyzed in duplicate. Results are mean (SD). Ab, antibody; CDC, complement-dependent cytotoxicity; DLBCL, diffuse large B-cell lymphoma; hIgG1, human immunoglobulin G1; MM, multiple myeloma; RFU, relative fluorescence units; SD, standard deviation.

We next checked whether isatuximab could induce deposition of C3b on the surface of target cells, one of the early steps in the complement cascade. C3b deposition was measured for all four MM and DLBCL cell lines naturally expressing CD38 at a high density (>250,000 molecules/cell), as well as for several selected cell lines with low parental CD38 expression that were also engineered to overexpress CD38. Isatuximab induced C3b deposition on the four MM and DLBCL cells with parental CD38 receptor density >250,000 molecules/cell. Interestingly, none of the cell lines with lower parental CD38 receptor density had C3b deposition on their surface after isatuximab treatment. After overexpression of CD38 >250,000 molecules/cell in these cell lines, C3b deposition occurred (Figure 8A). This result demonstrated that C3b deposition requires high CD38 expression on target cells.

### Complement Regulatory Proteins Limit Isatuximab-Induced CDC Activity

Since tumor cells may exploit complement regulatory proteins to prevent CDC, which might impact the efficacy of monoclonal antibodies employing CDC as part of their antitumor effect, we next examined the expression of the complement inhibitors CD46, CD55, and CD59 on the surface of MM and DLBCL cells. In CDC-resistant lines, one or more CDC inhibitor levels were >50,000 molecules/cell. Two CDC-susceptible cell lines, LP-1 and SUDHL-8, had expression levels of these CDC inhibitors <50,000 molecules/cell. These cell lines also had high surface density of CD38 (Figure 8B). Based on these data, we hypothesized that isatuximab-mediated CDC required both high-level expression of CD38 and low-level expression of CD46, CD55, and CD59.

To test our hypothesis, we first selected several cell lines with CD38 receptor density <100,000 molecules/cell and overexpressed CD38 at levels >250,000 molecules/cell. Two lines (U266.CD38^++^ and OCI-LY19.CD38^++^) that were susceptible to isatuximab-mediated CDC had expression levels of CDC inhibitors <50,000 molecules/cell. Three lines (NCI-H929.CD38^++^, RPMI-8226.CD38^++^, and JJN-3.CD38^++^) that were not susceptible to isatuximab-mediated CDC had CDC inhibitor surface densities above the 50,000 molecules/cell threshold (Figure 8C). Therefore, a low expression level of CDC inhibitors on target cells is also essential to enable isatuximab-mediated CDC. Importantly, overexpression of CD38 did not alter the expression of complement regulatory proteins, indicating that the high-level CD38 receptor density is insufficient to overcome the inhibitory effect of CDC inhibitors (Figure 8C).

### CD59 Inhibition Enables Isatuximab to Trigger Killing of MM Cells Otherwise Resistant to Isatuximab-Induced CDC

Thus far, we observed that isatuximab did not induce CDC in the U266, MOLP-8, or NCI-H929 cell lines; we postulated that this was because the expression level of CD38 was insufficient to trigger C3b deposition, levels of CDC inhibitors were too high, or a combination of these factors. U266 cells, which have low CD38 and CDC inhibitor expression, became sensitized to complement-dependent lysis when engineered to overexpress CD38. In contrast, the CDC-resistant MOLP-8 cell line expressed high CD38 levels but also exhibited high levels of the CDC inhibitor protein CD59. In this case, inhibition of CD59 resulted in CDC-mediated MOLP-8 cell lysis with isatuximab. For the NCI-H929 cell line, which expresses low levels of CD38 and high levels of CD59, both overexpression of CD38 and inhibition of CD59 were required to sensitize the cells to isatuximab-mediated CDC (Figure 8D). Collectively, these observations solidify the notion that inhibition of complement regulators may enhance isatuximab-mediated CDC in cells with high CD38 receptor density.

## Discussion

High expression of CD38 is consistently found on malignant plasma cells from MM patients (5, 6), as well as on malignant cells from other hematological cancers such as GCB-DLBCL (7). CD38 has become an appealing tumor-associated antigen to target with naked antibodies [including daratumumab, isatuximab, MOR202, and TAK-079 (37)], anti-CD38 chimeric antigen receptor (CAR)-T cells (38), and T-cell engagers (39, 40).

Isatuximab targets a specific epitope on CD38 that is distinct from the binding sites of other anti-CD38 monoclonal antibodies, including daratumumab. Isatuximab binding to the discontinuous conformational epitope on CD38 (14) activates several mechanisms leading to MM cell death. Preclinical studies have highlighted isatuximab's ability to induce Fc-dependent mechanisms such as ADCC, ADCP, and CDC (14, 20–22); Fc-independent mechanisms, including the induction of direct cytotoxicity in tumor cells (31); and inhibition of CD38 enzymatic activities (14), as well as wider immunomodulatory effects (20). We studied the Fc-dependent isatuximab mechanisms, as well as direct isatuximab-mediated NK cell activation, in the context of MM and DLBCL cell lines and MM patient samples, to gain mechanistic insights into isatuximab clinical activity.

We first characterized the expression of CD38 on immune cells derived from healthy donors as well as from MM patients, finding that the greatest proportion of CD38^+^ cells and highest expression levels were observed with NK cells and monocytes. Comparison of CD38 receptor density on immune cells from healthy donors and MM patients revealed that most immune cell lineages express comparable level of CD38, with the exception of a trend of elevated CD38 receptor density on NK and B cells from MM patients. In addition, bone marrow progenitor cells also express CD38 at a comparable level to that on NK cells and monocytes. Nevertheless, the CD38 density on healthy cells is significantly lower than that found on cells from patients with MM, consistent with previous observations (5, 6). Such a high level of expression of CD38 on MM cells suggests that there is an excellent therapeutic window for isatuximab targeting malignant cells via cytotoxic mechanisms, with minimal adverse effects on normal CD38-expressing immune cells anticipated (37).

We demonstrated that isatuximab can lyse different hematological malignant cells via Fc-dependent or -independent mechanisms. While MM cell lines with high levels of CD38 expression were more susceptible to isatuximab-mediated cytotoxicity through ADCC and ADCP, DLBCL cells were more sensitive to isatuximab-induced apoptosis and ADCC. Although the CD47-SIRPα pathway is critical in controlling phagocytic function of monocytes/macrophages (41) we found that only 10% of the effector THP1 cells used in the assay expressed a low level of SIRP1α, excluding the involvement of this pathway in the different responses between MM and DLBCL cells to isatuximab-mediated ADCP. Further investigation would be necessary to better understand mechanisms underpinning these differences.

Isatuximab's ability to induce ADCC and ADCP requires MM cells to express CD38 over a certain threshold level. We have shown that CD38 overexpression or increasing CD38 expression on MM cells using ATRA, a potent CD38 inducer, enhanced isatuximab-induced ADCC. It is likely that high CD38 receptor density on MM cells facilitated crosslinking of CD16 on NK cells in the presence of isatuximab, thereby stimulating NK cell function. These results provide a potential mechanism through which MM cells with high CD38 receptor density may be more effectively targeted for ADCC compared with low CD38 receptor density cells. Similarly, a recent study suggested that ATRA treatment of MM cell lines or patient samples has been shown to enhance the activity of daratumumab preclinically (35). Clinical investigation of the addition of ATRA to daratumumab in patients with RRMM suggested that patients who achieved a partial response or better with daratumumab monotherapy achieved an additional 7.8 months of disease control following the addition of ATRA and re-intensification of daratumumab therapy, compared with patients who did not respond initially (42).

NK cells play an important role in tumor surveillance; low NK cytotoxic activity has been associated with high risk of cancer (43), and NK cell-based therapeutic approaches hold promise for the treatment of MM patients (44). Enhancing the cytolytic activity of NK cells would enhance tumor cell killing. In addition to isatuximab-mediated NK cell activation via engaging high-level CD38-expressing tumor cells, isatuximab treatment of purified NK cells also led to direct NK cell activation and release of IFN-γ and TNF-α in the absence of target cells. Isatuximab-mediated NK cell activation via crosslinking of CD16 and CD38 led to efficient NK-mediated lysis of CD38^−^ target cells. This demonstrates that in addition to inducing NK cell-mediated ADCC, isatuximab can also directly activate NK cells and enhance NK cell-killing capacity. Interestingly, Viola et al. recently reported that daratumumab also directly activated NK cells (45). The authors further demonstrated that the daratumumab Fab fragment did not activate NK cells, indicating that without Fc receptor engagement, targeting CD38 alone cannot activate NK cells. Importantly, isatuximab-mediated NK activation is dependent on cross-linking CD16 and CD38 by the full-length isatuximab, whereas the monovalent daratumumab Fab, together with Fc fragment, seems to be sufficient to stimulate NK cells. This may be a potential mechanistic differentiation between isatuximab and daratumumab.

Signal transduction analysis showed that isatuximab treatment of NK-92 cells not only increased Src activation via synergy with the CD16 pathway, but also increased the phosphorylation of STAT3 and STAT5. Since an agonistic anti-CD16 antibody did not stimulate similar levels of STAT3 and STAT5 activation, the result indicated that, in addition to associating with CD16, CD38 might also interact with other receptors leading to STAT activation. It is known that IL-15 signaling induces the expression of CD137 on NK cells, which could contribute to NK cell proliferation and IFN-γ secretion (46). Interestingly, a recent study by Moreno and colleagues demonstrated that isatuximab can induce CD137 expression on NK cells (21). Collectively, it is plausible that isatuximab-mediated STAT phosphorylation could overlap, at least partially, with the IL-15 signaling pathway in driving CD137 expression.

NK-92 is a transformed cell line that does not express endogenous CD16. We compared NK-92 stable lines and primary NK cells in response to isatuximab stimulation in order to understand whether these stable cells exhibit a similar pattern to primary NK cells. Our result suggested that NK-92.CD16^V/V^ cells are more responsive to isatuximab stimulation and better mimic the activity of primary NK cells than NK-92.CD16^F/F^ cells. In addition, the different responses to isatuximab between NK-92.CD16^V/V^ and NK-92.CD16^F/F^ also indicates that the potency of isatuximab-mediated ADCC could be significantly improved via Fc engineering to enhance its CD16 binding. This strategy has been applied into the next-generation anti-CD38 antibody SAR442085 with higher affinity for the activating FcγRIIIa and FcγRIIa receptor. In preclinical studies, SAR442085 exhibits significantly improved dissociation constant, K_D_ to both CD16^F/F^ and CD16^V/V^, and enhanced ADCC by primary human NK cells (47).

Though NK cells play an important role in antitumor responses, their activation is regulated by several pathways. The PD-1/PD-L1 pathway is suggested to play a role in NK cell regulation in MM patients (48, 49). NK cells from MM patients, but not from healthy individuals, express PD-1, and MM cells express PD-L1 (25). Using a MM cell line/PBMC co-culture system, we demonstrated that MM cells were able to attenuate the cytolytic function of NK cells. It has been suggested previously that isatuximab targets both constitutive and inducible regulatory T cells (induced in the tumor microenvironment) in models of myeloma, resulting in increased effector cell response (50). Our experiments showed that during the co-culture, PD-1 expression was upregulated on NK cells and PD-L1 upregulated on monocytes, suggesting that PD-L1-expressing monocytes could mediate immune suppression of the NK cells. Indeed, *in vitro* blockade of the PD-1/PD-L1 pathway enhanced isatuximab-mediated ADCC of MM cells. Interestingly, we found that IL-6 drives PD-L1 expression on MM cells, in agreement with recent reports from preclinical models and patient samples in other cancers (51–54). The critical role of IL-6 in supporting the growth of MM cells in bone marrow is already well established (55), and our finding suggests that IL-6 could have an additional immunosuppressive effect in the bone marrow. The induction of PD-L1 expression on tumor cells, and possibly on other cell types, by IL-6 might represent an important immunosuppressive mechanism facilitating the escape of MM cells from immune surveillance. Therefore, blockade of IL-6 or PD-1/PD-L1 may have the potential to improve the therapeutic efficacy of isatuximab in the treatment of MM. Interestingly, lenalidomide downregulates PD-L1 on primary MM cells, which might enhance NK cell function in MM patients (25). Altogether, these findings have provided a rationale to explore PD-1 blockade to enhance isatuximab-induced ADCC of MM cells. Currently, a Phase 1/2 study in RRMM patients is ongoing to evaluate the safety, pharmacokinetics, and efficacy of isatuximab in combination with the anti-PD-1 antibody cemiplimab in RRMM patients (ClinicalTrials.gov: NCT03194867).

In addition to cell–cell contact-mediated immunosuppression, immunosuppressive cytokines such as TGF-β also play a critical role in dampening NK cell function (26). Using MM cell lines, we demonstrated that both recombinant TGF-β1 and TGF-β produced by MM cells suppressed the cytolytic activity of NK cells and isatuximab-induced ADCC. The inhibitory effect of TGF-β on NK cells was efficiently reversed by an anti-TGF-β neutralizing antibody. TGF-β1 suppresses granzyme B and perforin expression in NK cells, NK cell proliferation (unpublished observations). In addition, TGF-β produced by MM cells can inhibit later phases of osteoblast differentiation/maturation and suppress matrix mineralization. Thus, inhibition of TGF-β signaling could also enhance bone formation and suppress MM cell growth via mature osteoblast-dependent mechanism (56). Taken together, this suggests that combination treatment with isatuximab and an anti-TGF-β antibody would be an appealing approach not only to improve the therapeutic efficacy of isatuximab, but also to reduce bone destruction caused by myeloma cells.

Alongside the prominent role of NK cells, the complex role of monocytes in cancer include antitumoral effects through the phagocytosis of tumor cells (57). FcγR expression on macrophages, enabling ADCP, is known significantly contribute to the therapeutic effect of many antibodies approved to treat cancer (29). We showed that isatuximab treatment of primary monocytes or monocytic THP-1 cells enhanced the phagocytosis of CD38^−^ beads.

CDC has also been described as a mechanism of action of isatuximab (14, 20, 21). Isatuximab-mediated CDC was only observed in MM and DLBCL cell lines with high CD38 receptor density and low surface densities of the CDC inhibitors CD46, CD55, and CD59. We identified a relationship between the deposition of C3b, a component of the complement cascade, and CD38 receptor density, but found that high CD38 receptor density alone was insufficient for CDC lysis; no or low-level expression of CDC inhibitors was also required. Previously published experiments using a panel of MM cell lines (RPMI-8226, NCI-H929, MM.1S, and OPM-2) found that isatuximab induced C1q binding, without C3 deposition or an impact on cell survival (22). An explanation for these results is that CD38 receptor density on those cell lines were too low to induce CDC. The importance of continued characterization of cell lines, in this case the profiling of CDC inhibitor expression, is emphasized by our finding that the MOLP-8 cell line was resistant to isatuximab-induced CDC, in contrast to previous reports (14). However, MOLP-8 cells became susceptible to CDC on CD59 blockade, suggesting that CD59 expression levels may have changed. In fact, increased CD55 and CD59 levels have been detected on MM cells at the time of progression during daratumumab therapy, indicating that the treatment selected MM clones with high expression levels of CD55 and CD59 (58). This clinical observation, together with our data, suggested that upregulation of CDC inhibitors is a common mechanism for MM cells to escape antitumor immunity. This implies that upon single-agent isatuximab treatment in MM, ADCC and ADCP—rather than CDC—are likely to be the major mechanisms of action. This is consistent with previous preclinical findings that have highlighted the importance of ADCC (14, 21). Development of resistance to daratumumab has been associated with upregulation of CD55 and CD59, since their expression increased on patient-derived MM cells at the time of disease progression (58). In contrast, the lower dependence of isatuximab on CDC suggests that the development of similar resistance in isatuximab-treated patients would be unlikely. In the future, new antibody formats with enhanced CDC functions could overcome resistance to CDC in the treatment of MM. A hexamerization-enhanced anti-CD38 antibody has recently demonstrated improved CDC activity against MM cell lines and patient samples compared with daratumumab, while retaining comparable ADCC and ADCP activity (59). In addition, CD38-specific biparatopic antibodies have demonstrated CDC activity beyond that of daratumumab (60), representing another promising therapeutic strategy.

While we believe that these studies have effectively characterized the mechanisms of antitumor activity key to the initial efficacy of isatuximab, it seems likely that additional immunomodulatory effects become important during treatment. Firstly, it has been suggested (50) that isatuximab targets and downregulates both constitutive and inducible regulatory T cells in the tumor microenvironment of myeloma models, resulting in increased effector cell response. Secondly, results from a Phase 2 study of isatuximab monotherapy demonstrated a statistically significant increase in T-cell receptor clonality following three and five cycles of treatment, compared with the start of treatment (61), suggesting that isatuximab may improve the host antitumor immune response. Finally, a small immunomonitoring study in patients with RRMM treated with isatuximab revealed that patients with pre-existing anti-myeloma immune responses developed additional immune responses against CD38 in response to isatuximab treatment, whereas patients with few pre-existing antitumor antibodies developed no new serological responses to treatment. These data suggest the possibility of an adaptive antitumor immunity in response to isatuximab (62).

In conclusion, we characterized multiple mechanisms of action of isatuximab relevant to its therapeutic activity in MM. Our observations support ADCC as a key effector mechanism for isatuximab, which is dependent on the expression level of CD38 and susceptible to suppression by the PD-1/PD-L1 pathway and TGF-β secretion by MM cells. Furthermore, we established that isatuximab can directly activate NK cells and stimulate NK cell-mediated cytotoxicity through crosslinking CD38 and CD16. Similarly, we have shown that isatuximab directly increases phagocytic activity of monocytes in a dose-dependent manner. The low proportion of cell lines and MM patient samples deemed potentially susceptible to isatuximab-induced CDC reflects the anticipated minor contribution of CDC to isatuximab clinical activity. Following the success of the first Phase 3 isatuximab clinical trial, evaluation of isatuximab mechanisms in samples from isatuximab-treated patients represents an exciting future prospect. In addition, mechanistic insights gained from this study could be used to design novel combination regimens, aiming to optimize the treatment of MM patients with currently unmet needs.

## Data Availability Statement

The raw data supporting the conclusions of this article will be made available by the authors, without undue reservation, to any qualified researcher.

## Author Contributions

ZS, AW, SS, GY, RG, and ES performed the experiments. JT performed bioinformatic analyses. LW, Z-YY, and AF generated tool antibodies. WP-C performed CD38 expression analysis on MM cells from patients. Y-TT and KA collected blood samples from MM patients and provided experimental designs. CZ and FA designed the studies. DW, KB, CZ, FA, and MC analyzed and interpreted the results. All authors participated in writing and critically reviewing the manuscript and approved the final version for submission.

## Conflict of Interest

CZ, ZS, AW, RG, JT, ES, LW, Z-YY, WP-C, AF, DW, KB, and MC: Employment – Sanofi. KA: Consultancy – Celgene, Millennium, Takeda, Bristol-Myers Squibb, Gilead, Sanofi, Tolero Pharmaceuticals, Precision Biosciences; Scientific Founder – OncoPep, C4 Therapeutics. SS, GY, and FA: Employment – Formerly employed by Sanofi. The remaining author declares that the research was conducted in the absence of any commercial or financial relationships that could be construed as a potential conflict of interest.

## Abbreviations

ADCC: antibody-dependent cellular cytotoxicity
ADCP: antibody-dependent cellular phagocytosis
ATCC: American Type Culture Collection
ATRA: all-trans retinoic acid
BSA: bovine serum albumin
CCLE: Cancer Cell Line Encyclopedia
CDC: complement-dependent cytotoxicity
DLBCL: diffuse large B-cell lymphoma
DSMZ: Deutsche Sammlung von Mikroorganismen und Zellkulturen
FBS: fetal bovine serum
Fc: fragment crystallizable
FITC: fluorescein isothiocyanate
GCB: germinal center B-like
Gzm: granzyme
hIgG1: human immunoglobulin G1
HSRRB: Health Science Research Resources Bank
IFN: interferon
IgG: immunoglobulin G
IL: interleukin
Isa^*^: isatuximab mutant unable to bind to CD38
MM: multiple myeloma
MSD: Meso Scale Diagnostics
MSD: mesoscale discovery
NK: natural killer
NT: not treated
PBMC: peripheral blood mononuclear cell
PBS: phosphate-buffered saline
PD-1: programmed cell death-1
PD-L1: programmed cell death-ligand 1
PMA: phorbol myristate acetate
RD: receptor density
RFU: relative fluorescence units
RRMM: relapsed and refractory multiple myeloma
SD: standard deviation
TGF: transforming growth factor
TNF: tumor necrosis factor
Treg: regulatory T cell.
