# Supplementary material for: Isatuximab Acts Through Fc-Dependent, Independent, and Direct Pathways to Kill Multiple Myeloma Cells
Source: Front Immunol. 2020 Aug 14;11:1771. doi: 10.3389/fimmu.2020.01771 (PMC7457083; doi:10.3389/fimmu.2020.01771)
Supplement: Supplementary file 1 [file Data_Sheet_1.pdf]

Isatuximab Acts Through Fc-Dependent, Independent, and Direct Pathways to Kill Multiple Myeloma Cells

Chen Zhu[1*], Zhili Song[1], Anlai Wang[1], Srimathi Srinivasan[1], Guang Yang[1], Rita Greco[1], Joachim Theilhaber[1], Elvis Shehu[1], Lan Wu[2], Zhi-Yong Yang[2], Wilfried Passe-Coutrin[3], Alain Fournier[4], Yu-Tzu Tai[5], Kenneth C. Anderson[5], Dmitri Wiederschain[1], Keith Bahjat[1], Francisco J. Adrián[1†‡] and Marielle Chiron[1†]

[1]Sanofi Oncology, Cambridge, MA, United States, [2]Sanofi Research and Development, Sanofi North America, Cambridge, MA, United States, [3]Sanofi R&D, Biomarkers and Clinical Bioanalyses, Paris, France, [4]Sanofi R&D, Tumor-targeted immuno-modulation I, Vitry-sur-Seine, France, [5]Jerome Lipper Multiple Myeloma Center, LeBow Institute for Myeloma Therapeutics, Dana-Farber Cancer Institute, Harvard Medical School, Boston, MA, United States

*Correspondence: Chen Zhu
Chen.Zhu@sanofi.com

[†]Contributed equally

[‡]Affiliation at time of study, currently HiFiBiO Therapeutics, Cambridge, MA, United States

SUPPLEMENTARY MATERIAL

Supplementary Table 1. CCLE (1) cell lines used in this study.

CCLE cell line	Indication
BJAB	Diffuse large B cell lymphoma
JURKAT	Acute lymphoblastic T cell leukemia
Daudi	Burkitt lymphoma
EJM	Plasma cell myeloma
HBL-1	Diffuse large B cell lymphoma
HT	B cell lymphoma (unspecified)
HuNS1	Plasma cell myeloma
JJN-3	Plasma cell myeloma
KARPAS-620	Plasma cell myeloma
KMS-11	Plasma cell myeloma
KMS-12-BM	Plasma cell myeloma
KMS-20	Plasma cell myeloma
KMS-26	Plasma cell myeloma
L-363	Plasma cell myeloma
LP-1	Plasma cell myeloma
MM.1S	Plasma cell myeloma
MOLP-2	Plasma cell myeloma
MOLP-8	Plasma cell myeloma
NCI-H929	Plasma cell myeloma
OCI-LY10	Diffuse large B cell lymphoma
OCI-LY19	Diffuse large B cell lymphoma
OCI-LY3	Diffuse large B cell lymphoma
OPM-2	Plasma cell myeloma
RPMI-8226	Plasma cell myeloma
SK-MM-2	Plasma cell myeloma
SUDHL-10	Diffuse large B cell lymphoma
SUDHL-4	Diffuse large B cell lymphoma
SUDHL-6	Diffuse large B cell lymphoma
SUDHL-8	Diffuse large B cell lymphoma
TMD-8	Diffuse large B cell lymphoma
U266	Plasma cell myeloma

U2932	Diffuse large B cell lymphoma
U2973	Diffuse large B cell lymphoma
WSU-DLCL2	Diffuse large B cell lymphoma

CCLE, Cancer Cell Line Encyclopedia.

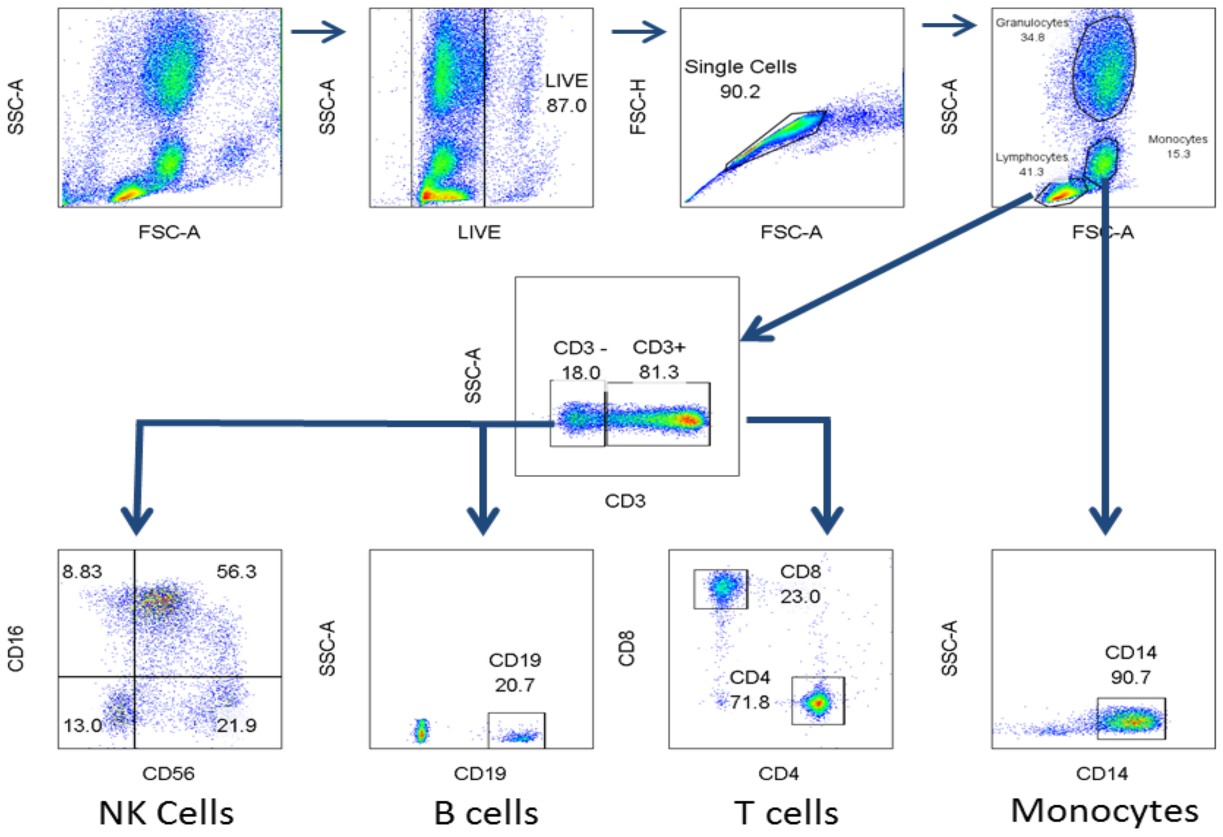

Supplementary Figure 1. Gating strategy for granulocytes, NK cells, B cells, monocytes, and CD4 and CD8 T cells in human PBMCs.

FSC-A, forward scatter area; FSC-H, forward scatter height; NK, natural killer; PBMC, peripheral blood mononuclear cell; SSC-A, side scatter area

A

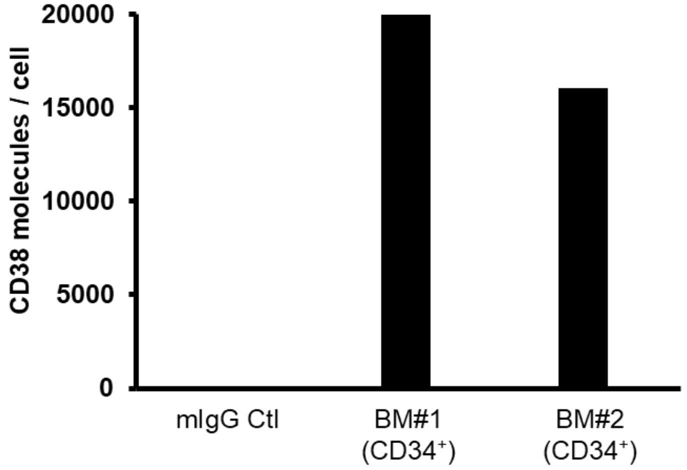
CD38 molecules / cell
20000
15000
10000
5000
0
mIgG Ctl
BM#1
(CD34+)
BM#2
(CD34+)

B

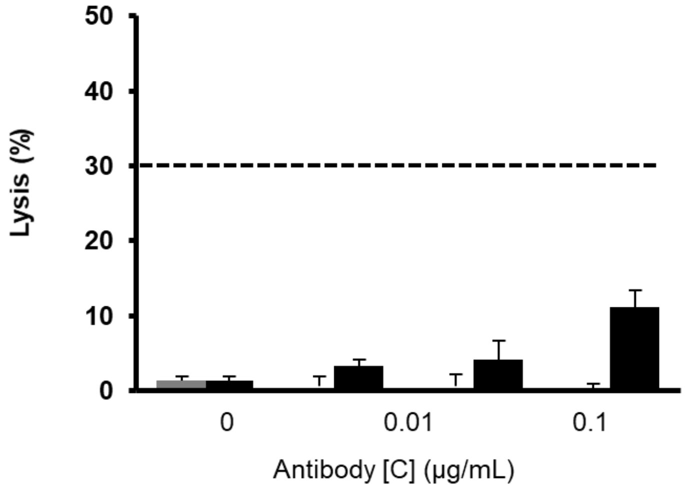
Lysis (%)
50
40
30
20
10
0
0
0.01
0.1
Antibody [C] (µg/mL)

C

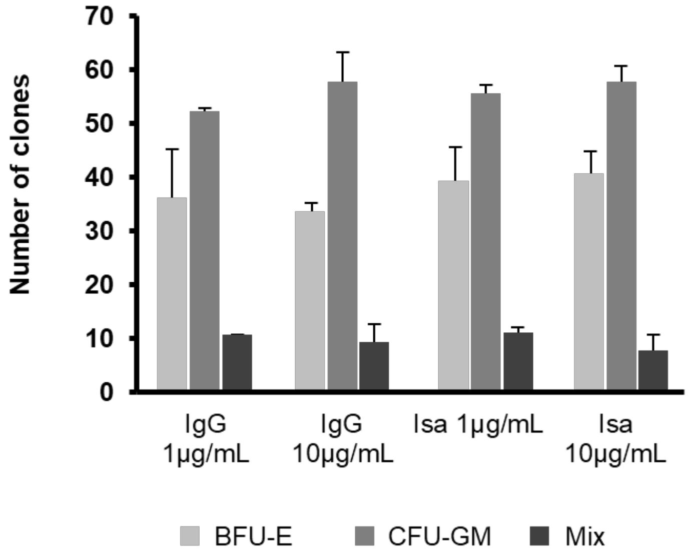

D

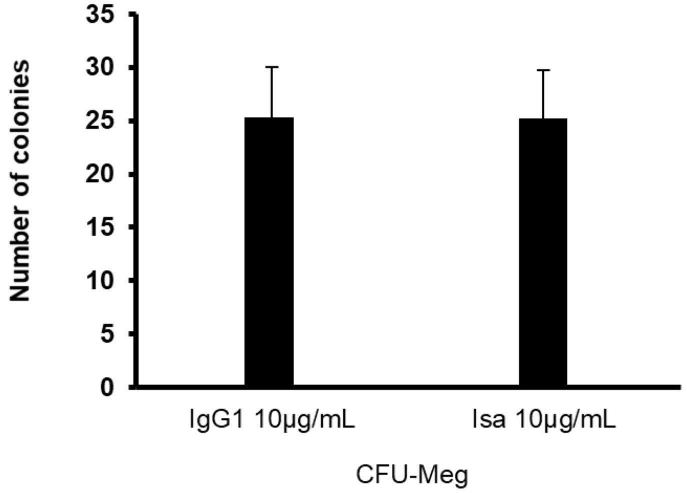

Supplementary Figure 2. Effect of isatuximab on human bone marrow $CD34^+$ progenitor cells. (**A**) Human bone marrow $CD34^+$ progenitor cells from two donors were stained with equal amount of mouse IgG1, k isotype control antibody (clone MOPC-21) or mouse anti-human CD38 antibody (clone AT13/5). The surface density of CD38 was quantified by QIFIKIT and FACS analysis. (**B**) Purified BM stem cells were tested for their sensitivity to isatuximab-induced ADCC. The ratio of effector cells (NK-92.$CD16^{V/V}$ cells) and target cells (BM stem cells) was 5:1. The percentage of ADCC

lysis was measured by calcein AM release from the stem cells. Black dashed line indicates a threshold for isatuximab induced ADCC in the assay.

(**C**) 5×10^2 purified BM stem cells were cultured in 35 mm culture dishes for 14 to 16 days with the presence of indicated concentrations of isatuximab or IgG1 isotype control in MethoCult™ H4034 Optimum medium. BFU-E, CFU-GM and mix colonies were quantified under a microscopy. Each treatment was analyzed in duplicate. Results are Mean (SD). (**D**) 5×10^3 purified BM stem cells were cultured in Double Chamber Slides for 10 to 12 days at 37°C with the presence of indicated concentrations of isatuximab or IgG1 isotype control in MegaCult™-C Medium with Cytokines, followed by applying anti-CD41 immunostaining process. CFU-Meg colonies then were quantified under a microscopy. Each treatment was analyzed in duplicate. Results are Mean (SD).

BM, bone marrow; ADCC, antibody-dependent cellular cytotoxicity; BFU-E, furst-forming unit-erythroid; CFU-GM, colony-forming unit-granulocyte macrophage; CFU-Meg, colony-forming unit megakaryocyte; IgG1, immunoglobulin G1; SD, standard deviation

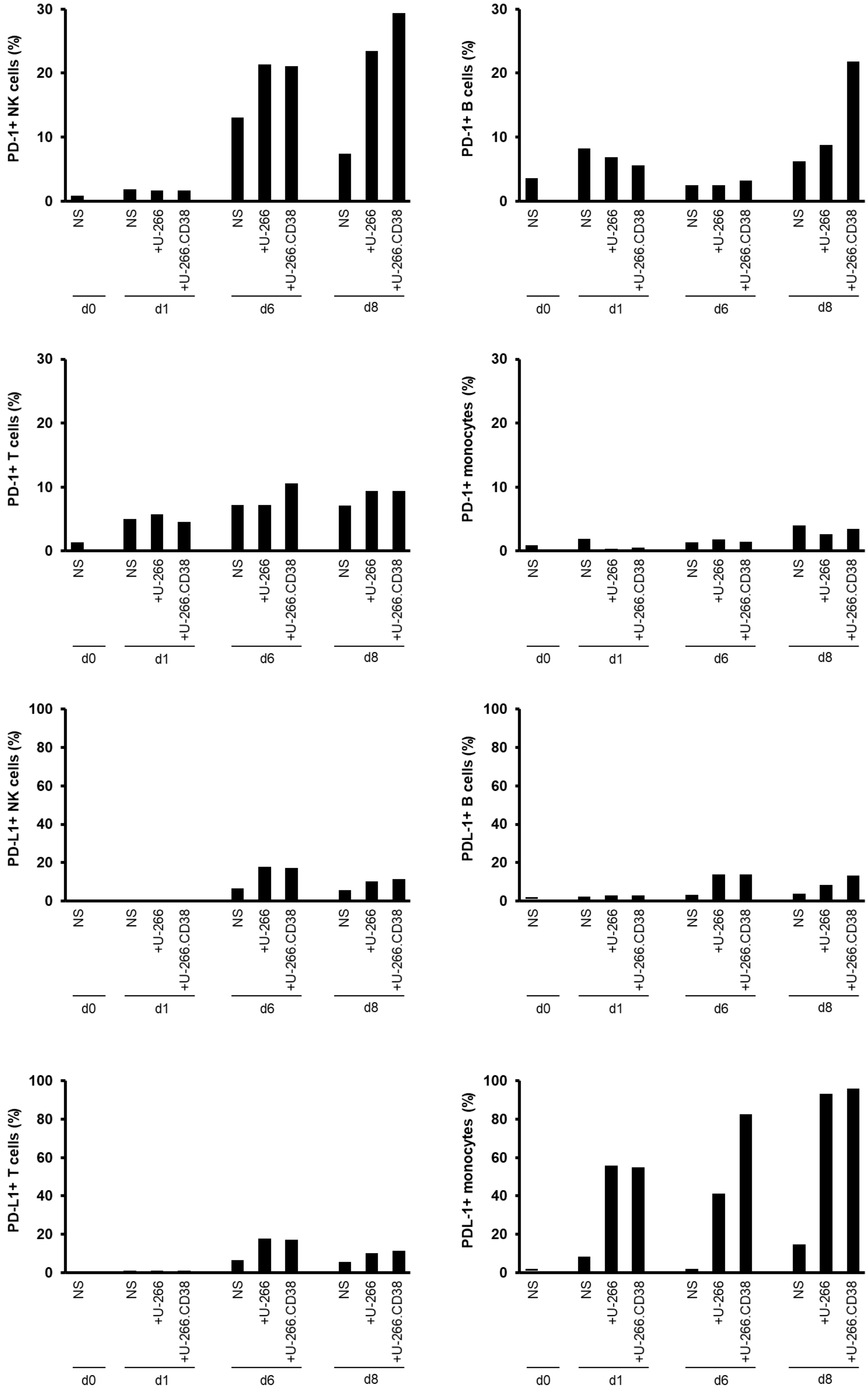
PD-1+ NK cells (%)
PD-1+ B cells (%)
PD-1+ T cells (%)
PD-1+ monocytes (%)
PD-L1+ NK cells (%)
PDL-1+ B cells (%)
PD-L1+ T cells (%)
PDL-1+ monocytes (%)
0
10
20
30
40
60
80
100
NS
+U-266
+U-266.CD38
d0
d1
d6
d8

Supplementary Figure 3. Induction of PD-1 and PD-L1 expression on immune cells after co-culture with MM cells. 5×10^6 PBMCs cultured with or without 5×10^5 U266 cells overexpressing CD38 (U266.CD38^{++}) in ultra-low attachment 6-well plates were analyzed for expression of PD-1 and PD-L1 on CD3$^+$ (T cells), CD14$^+$ (monocytes), and CD56$^+$ (NK cells) cells by flow cytometry on days 0, 1, 6, and 8 during the co-culture. Each assay was performed four to six times, using samples from six donors.

MM, multiple myeloma; NK, natural killer; PBMC, peripheral blood mononuclear cell; PD-1, programmed cell death-1; PD-L1, programmed cell death-ligand 1.

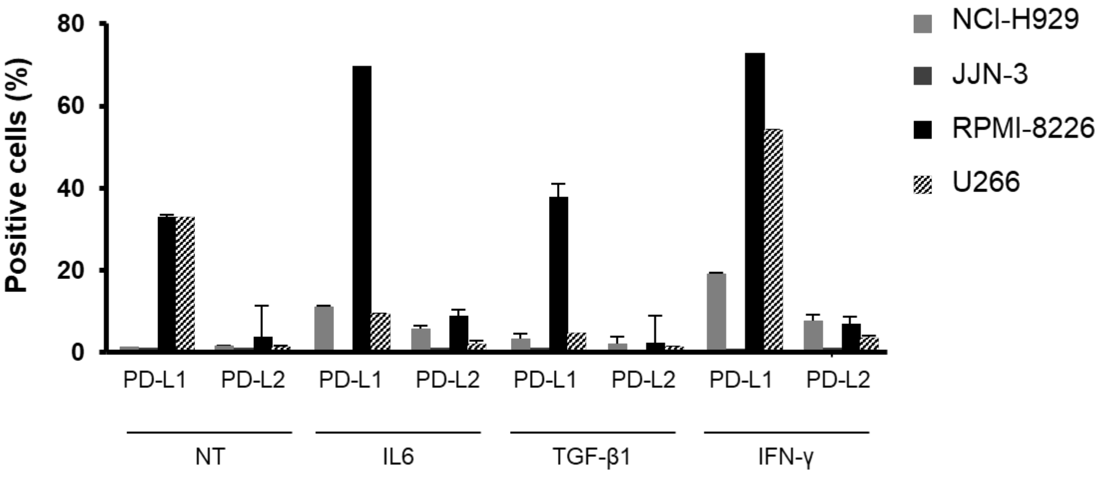

Supplementary Figure 4. Effect of PD-L1/PD-1 interaction on isatuximab ADCC activity. 2×10^6 MM cells were treated with 10 ng/ml of IL-6 or TGF-β1 or 100 ng/ml of IFN-γ at 37ºC for 48 hours and the expression of PD-L1 and PD-L2 was quantified by flow cytometry. Experiments were performed at least three times in triplicate for each criterion.

ADCC, antibody-dependent cellular cytotoxicity; IFN-γ, interferon-gamma; IL-6, interleukin-6; NT, not treated; PD-1, programmed cell death-1; PD-L1, programmed cell death-ligand 1; PD-L2, programmed cell death-ligand 2; TGF-β, transforming growth factor-beta.

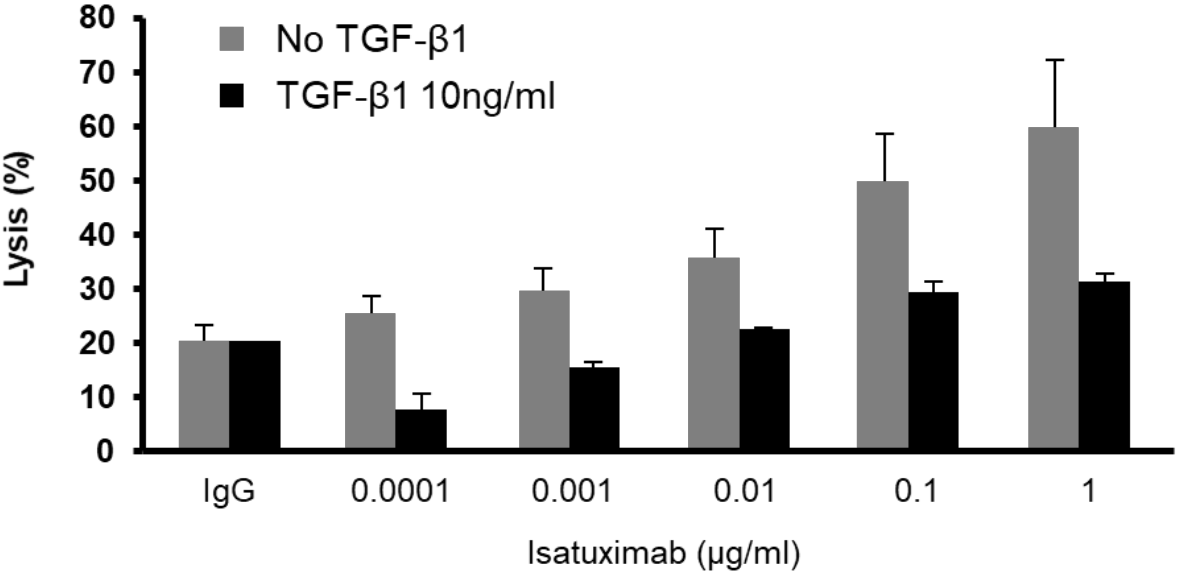

Supplementary Figure 5. Pre-conditioning of NK-92.CD16$^{V/V}$ cells with recombinant TGF-β1 reduces isatuximab-induced ADCC. NK-92.CD16$^{V/V}$ cells were treated with 10 ng/ml of TGF-β1 at 37°C for 90 hours and used as effector cells in isatuximab-mediated killing of MOLP-8 target cells and ADCC quantified by calcein AM release. The experiment was performed in technical triplicate.

ADCC, antibody-dependent cellular cytotoxicity; IgG, immunoglobulin G; TGF-β1, transforming growth factor-beta 1.

References

1. Broad Institute. Cancer Cell Line Encyclopedia. Available from: https://portals.broadinstitute.org/ccle.
